# Type 2 diabetes mellitus and antibiotic-resistant infections: a systematic review and meta-analysis

**DOI:** 10.1136/jech-2020-216029

**Published:** 2021-07-29

**Authors:** Rodrigo M Carrillo-Larco, Cecilia Anza-Ramírez, Giancarlo Saal-Zapata, David Villarreal-Zegarra, Jessica Hanae Zafra-Tanaka, Cesar Ugarte-Gil, Antonio Bernabé-Ortiz

**Affiliations:** 1 Department of Epidemiology and Biostatistics, Imperial College London School of Public Health, London, UK; 2 CRONICAS Centre of Excellence in Chronic Diseases, Universidad Peruana Cayetano Heredia, Lima, Peru; 3 Hospital Nacional Guillermo Almenara Irigoyen – EsSalud, Lima, Peru; 4 Facultad de Medicina Alberto Hurtado, Universidad Peruana Cayetano Heredia, Lima, Peru; 5 Universidad Peruana Cayetano Heredia Instituto de Medicina Tropical Alexander von Humboldt, Lima, Peru; 6 Department of Clinical Research, London School of Hygiene and Tropical Medicine, London, UK; 7 Universidad Cientifica del Sur, Lima, Peru

**Keywords:** nutritional sciences, infections, public health

## Abstract

**Background:**

Type 2 diabetes mellitus (T2DM) has been associated with infectious diseases; however, whether T2DM is associated with bacterial-resistant infections has not been thoroughly studied. We ascertained whether people with T2DM were more likely to experience resistant infections in comparison to T2DM-free individuals.

**Methods:**

Systematic review and random-effects meta-analysis. The search was conducted in Medline, Embase and Global Health. We selected observational studies in which the outcome was resistant infections (any site), and the exposure was T2DM. We studied adult subjects who could have been selected from population-based or hospital-based studies. I^2^ was the metric of heterogeneity. We used the Newcastle-Ottawa risk of bias scale.

**Results:**

The search retrieved 3370 reports, 97 were studied in detail and 61 (449 247 subjects) were selected. Studies were mostly cross-sectional or case–control; several infection sites were studied, but mostly urinary tract and respiratory infections. The random-effects meta-analysis revealed that people with T2DM were twofold more likely to have urinary tract (OR=2.42; 95% CI 1.83 to 3.20; I^2^ 19.1%) or respiratory (OR=2.35; 95% CI 1.49 to 3.69; I^2^ 58.1%) resistant infections. Although evidence for other infection sites was heterogeneous, they consistently suggested that T2DM was associated with resistant infections.

**Conclusions:**

Compelling evidence suggests that people with T2DM are more likely to experience antibiotic-resistant urinary tract and respiratory infections. The evidence for other infection sites was less conclusive but pointed to the same overall conclusion. These results could guide empirical treatment for patients with T2DM and infections.

## Introduction

With a large burden in terms of morbidity, mortality, disability and economic costs,^1–4^ type 2 diabetes mellitus (T2DM) is a global health problem disproportionally affecting low-income and middle-income countries (LMICs).^1–4^ While much of the research about T2DM has focused on its determinants, consequences and complications regarding non-communicable diseases, T2DM as a risk and prognostic factor for infectious—communicable—diseases has gained attention lately.^5–8^ In this relatively novel field—T2DM and infectious diseases—antibiotic resistance remains understudied, though it carries a large disease burden globally and in LMICs.^9–13^

Large studies about T2DM and antibiotic resistance have focused on one pathogen or colonisation (rather than infection).^14^ Moreover, there appears to be discrepancies on whether T2DM is a risk factor for infections with antibiotic-resistant bacteria depending on the infection site. For example, some authors have suggested that T2DM is not an independent risk factor for urinary tract infections with resistant bacteria;^15^ however, for community-acquired intra-abdominal infections, T2DM has been described as a potential risk factor.^16^

The large burden of T2DM,^1–4^ paired with its potential role as a risk and prognostic factor for infectious diseases,^5–8^ along with the global issue of antibiotic resistance,^9–13^ call to thoroughly study whether people with T2DM are at higher risk of infections with resistant bacteria. This knowledge may guide empirical treatment, with a subsequent positive impact on T2DM patients who would recover faster from infections, while also reducing the burden of antibiotic-resistant bacteria by prescribing more accurate treatments. Consequently, to understand whether T2DM is a risk factor for infections with resistant bacteria, in comparison to non-resistant infections, and whether there are any differences depending on the infection site, we conducted a systematic review of the scientific literature and a random-effects meta-analysis.

## Methods

### Protocol

We aimed to ascertain if people with T2DM, in comparison to otherwise healthy individuals, were more likely to experience a resistant infection rather than an infection with non-resistant bacteria. We hypothesised that, in comparison to T2DM-free individuals, people with T2DM who experience an infection, this is more likely to be an antibiotic-resistant infection. We conducted a systematic review and random-effects meta-analysis. The search strategies as well as the screening and selection processes were planned in advance and not modified afterwards. This manuscript adheres to the Preferred Reporting Items for Systematic Reviews and Meta-Analyses recommendations (online supplemental table 1).^17^

10.1136/jech-2020-216029.supp1 Supplementary data

### Eligibility criteria

The inclusion criteria were: (i) the original studies could have been conducted in the community (eg, population-based random sampling) or in healthcare facilities (eg, consecutive patients in a clinic); (ii) original studies followed an observational design with a comparison group (eg, cross-sectional, case–control or prospective/retrospective cohorts); (iii) among the study participants, there were people with T2DM and without T2DM (comparison group); and (iv) the outcome was an infection with an antibiotic-resistant bacteria, as defined by each original report. Only studies with adult subjects were included.

The exclusion criteria were: (i) reports looking at colonisation with antibiotic-resistant bacteria (rather than infection) and (ii) research in which people with T2DM and other major or long-lasting conditions were studied, these included: neoplasms, tuberculosis, HIV/AIDS, bedridden, cerebral palsy, Alzheimer’s disease and vector-borne diseases (eg, malaria or dengue). We excluded these groups of patients because we targeted people with T2DM without any additional risk factors that could make them more likely to experience resistant infections. Studies looking at infections of viral aetiology were also excluded.

### Information sources

We used OVID to search in Medline, Embase and Global Health. The search was conducted in February 2020. The search was restricted to studies with human beings; no further restrictions were included. The search terms we used are available in online supplemental table 2.

### Search and study selection

The search results were downloaded to EndNote where duplicates were excluded. We then uploaded the results to Rayyan, an online open-access tool to conduct systematic reviews.^18^ Titles and abstracts were screened by two reviewers independently (RMC-L and AB-O); the same two reviewers studied the complete text of the reports selected in the screening phase. Discrepancies were solved by consensus between these two reviewers.

### Data collection

Data from the selected reports were extracted by two groups of reviewers independently (CA-R and GS-Z as well as JHZ-T and DV-Z). Discrepancies were solved by consensus within and between these pairs of reviewers, or by consensus with a third party (RMC-L). We designed a data extraction form which was agreed on by consensus among all the reviewers; this form was not modified during data collation.

The data extraction form included study characteristics (eg, year and country of data collection, whether population-based or hospital-based, and study design), and characteristics of the study population (eg, mean age, proportion of men and T2DM proportion). We also extracted information about the infection: infection site, as well as frequency of people with and without T2DM with a resistant infection; when available, we also collated information about the specific pathogens studied and antibiotics tested. When original studies reported an association metric (and not only proportions), we extracted those as presented in the reports (eg, OR, prevalence ratio or risk ratio). Of note, the meta-analysis is based on adjusted association metrics only; these were prioritised over unadjusted estimates because adjusted estimates would reflect solid and less biased evidence.

### Risk of bias in individual studies

We assessed the risk of bias of individual studies with the Newcastle-Ottawa Scale, a tool to assess quality of non-randomised studies.^19^ For case–control and cohort studies, we used the specific scales for these study designs. For cross-sectional studies, however, we used the same scale as for cohort studies applying all relevant criteria. This tool ranks studies with stars, where the more stars the less risk of bias the study shows. This process was conducted by two reviewers independently (CA-R and GS-Z; JHZ-T and DV-Z), and discrepancies were solved by consensus among them or after further consideration with a third party (RMC-L).

### Synthesis of results

First, we narratively presented the collated information for all the selected studies. Before data analysis, we decided to conduct a meta-analysis if there were at least four estimates per infection site (ie, at least four original publications or results). In the meta-analysis, we combined all study designs (cross-sectional, prospective and case–control studies), but only estimates of the same infection site (eg, urinary tract infections). We also combined estimates regardless of the specific bacteria or antibiotic studied. We only pooled adjusted estimates available in the original reports. We conducted a random-effects meta-analysis in Stata V.15 (StrataCorp) with the DerSimonian and Laird method. Pooled estimates are reported as OR and 95% CIs. Because of the limited number of studies with similar specific characteristics, it was no possible to conduct subgroup analyses (eg, by resistant bacteria or community-based vs hospital-based studies); however, when possible, pooled estimates were reported separately by cross-sectional/cohort or case–control studies. Finally, the I^2^ was reported as a metric of heterogeneity. A priori, we expected heterogeneity across reports because they studied different populations, were conducted in unique settings, and followed different methods.

## Results

### Study characteristics

**Figure 1:**
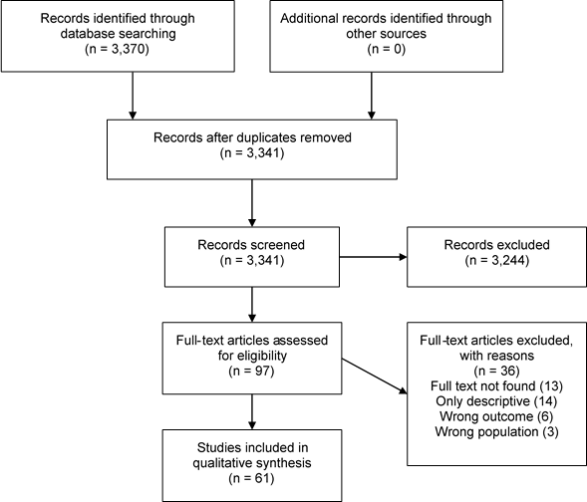
Study selection process. Depending on the outcome and subgroup analysis, the number of studies included in the meta-analysis varied. Therefore, the number of selected reports for quantitative synthesis is not reported in this figure but reported in the text.

The search yielded 3370 results, we screened 3341 and studied in detail 97 reports; finally, 61 (449 247 subjects) reports were included in the review (figure 1, tables 1 and 2). Reports were informed by data collected since 1989^20^–2017.^21 22^ The studies were conducted in Argentina,^16^ Bangladesh,^23 24^ Belgium,^25^ Brazil,^26 27^ Canada,^28 29^ China,^21 30–32^ Egypt,^33^ Finland,^34^ France,^35–37^ Germany,^37^ Greece,^38^ Guyana,^22^ India,^39^ Italy,^37 40^ Israel,^28 41–43^ Japan,^44^ Korea,^45 46^ Madagascar,^47^ Nepal,^48^ Netherlands,^15^ Norway,^49^ Pakistan,^50^ Poland,^51^ Singapore,^20 52 53^ Spain,^37 54–57^ Sweden,^34 58^ USA,^37 59–75^ Taiwan^76 77^ and Thailand.^78^

**Table 1:** Characteristics of cross-sectional and cohort reports

Author	Country	Year of data collection	Study design	Sample based	Sample size	Age	% men
Bailey *et al* 63	USA	2010	Cross-sectional	Hospital-based	222	NI	18.47
Baillargeon *et al* 59	USA	2000	Cohort	Captive population-based	299 179	NI	88.84
Benenson *et al* 33	Egypt	2001	Cross-sectional	Hospital-based	131	69.00±25.00	49.00
Bonadio *et al* 40	Italy	1999	Cross-sectional	Hospital-based	1321	72.96±22.00	31.00
Chen *et al* 76	Taiwan	2010	Cross-sectional	Hospital-based	420	63.5±13.97	24.60
Chiquet *et al* 35	France	2008	Cross-sectional	Hospital-based	68	76.20±11.40	44.12
Chiu *et al* 77	Taiwan	2015	Cross-sectional	Hospital-based	457	71.9	32.95
Chong *et al* 52	Singapore	2000	Cross-sectional	Hospital-based	100	59.36	NI
Chong *et al* 52	Singapore	2009	Cross-sectional	Hospital-based	98	49.06	NI
Ho *et al* 53	Singapore	2016	Cross-sectional	Hospital-based	299	60.80±17.30	13.38
Jääskeläinen *et al* 34	Finland and Sweden	2010	Cross-sectional	Hospital-based	390	66.14±18.60	58.21
Kistler *et al* 60	USA	2010	Cross-sectional	Hospital-based	815	49.54	NI
Kurup *et al* 22	Guyana	2017	Cross-sectional	Hospital-based	183	55.84±15.68	50.82
Laupland *et al* 29	Canada	2003	Cohort	Hospital-based	1542	61.70±22.19	62.00
Levin *et al* 28	Canada and Israel	2003 and 2005	Cross-sectional	Hospital-based	423	56.00±21.97	60.28
Libert *et al* 25	Belgium	2003	Cross-sectional	Hospital-based	154	63.2±56.9	52.60
Liu *et al* 21	China	2017	Cross-sectional	Hospital-based	456	64.00±15.72	72.15
Lye *et al* 20	Singapore	1989	Cross-sectional	Hospital-based	348	NI	NI
Madaras-Kelly *et al* 64	USA	2006	Cross-sectional	Hospital-based	375	71.20±12.40	98.67
Malmartel and Ghasarossian^36^	France	2014	Cross-sectional	Hospital-based	1119	59.14±19.96	26.22
Micek *et al* 37	USA, France Germany, Italy and Spain	2002	Cross-sectional	Hospital-based	740	59.47±16.61	67.97
Nakamura *et al* 44	Japan	2003	Cross-sectional	Hospital-based	740	NI	NI
Nuñez *et al* 16	Argentina	2016	Cross-sectional	Hospital-based	119	54±21	41.18
Papazafiropoulou *et al* 38	Greece	2008	Cross-sectional	Hospital-based	1244	72.30±12.60	31.35
Patolia *et al* 65	USA	2014	Cross-sectional	Hospital-based	177	±	57.00
Pinheiro *et al* 26	Brazil	2004 and 2007	Cohort	Hospital-based	45	42.00±14.50	68.89
Ramos Lázaro *et al* 54	Spain	2011	Cross-sectional	Hospital-based	552	66.00±17.00	100.00
Randrianirina *et al* 47	Madagascar	2007	Cross-sectional	Hospital-based	651	37.60±19.64	59.40
Rogers *et al* 61	USA	2003	Cross sectional and Cohort	Captive population-based	56 182	NI	30.79
Romaniszyn *et al* 51	Poland	2010	Cohort	Captive population-based	193	79.90±11.60	39.90
Sherchan *et al* 48	Nepal	2015	Cross-sectional	Hospital-based	645	NI	30.70
Terpenning *et al* 62	USA	1990	Cross-sectional	Hospital-based	551	64.40±0.50	98.37
Wu *et al* 30	China	2010	Cross-sectional	Hospital-based	136	67.00	31.62
Zhang *et al* 31	China	2016	Cross-sectional	Hospital-based	365	66.60±1.40	67.67

For an expanded version of this table (ie, containing more details about the study population in each original report) refer to online supplemental tables 3 and 6.

NI, no information available.

**Table 2:** Characteristics of case–control reports

Author	Country	Year of data collection	Sample based	Participant selection		Sample size		Age		% men	
				Case	Control	Case	Control	Case	Control	Case	Control
Anesi *et al* 66	USA	2012	Hospital-based	Consecutive patients	Random	151	151	58.67±17.96	46.33±28.44	28.48	12.58
Apisarnthanarak *et al* 78	Thailand	2004	Hospital-based	Random	Random	46	46	58.25±16.51	56.5±14.47	28.26	28.26
Aswani *et al* 39	India	2011	Hospital-based	NI	NI	181	124	60.2±13.76	53.47±18.56	45.86	41.94
Borer *et al* 42	Israel	2007	Hospital-based	Consecutive patients	Not clear	42	464	60.7±55.30	62.8±55	NI	NI
Briongos-Figuero *et al* 55	Spain	2010	Hospital-based	NI	Random	97	103	78±14	77±13	45.40	31.10
Chitnis *et al* 71	USA	2010	Hospital-based	Consecutive patients	Not clear	34	34	68.67±34.80	69.67±42.56	38.00	65.00
Colodner *et al* 41	Israel	2005	Hospital-based	Consecutive patients	Consecutive patients	150	150	63.75±14.20	56±14	24.70	10.70
Colodner *et al* 43	Israel	NI	Hospital-based	Consecutive patients	Consecutive patients	128	183	61.5±22.90	46.4±24.90	35.90	13.70
Dan *et al* 72	USA	2012	Hospital-based	Consecutive patients	Consecutive patients	143	681	67.7±14.23	65.3±18.60	59.00	44.00
Ashley *et al* 73	USA	1997	Hospital-based	Consecutive patients	Consecutive patients	64	79	67.30	61.80	45.30	69.60
García-Tello *et al* 57	Spain	2012	Hospital-based	Consecutive patients	Consecutive patients	416	1108	72.60±20.50	54.70±29.80	36.10	21.50
Hayakawa *et al* 67	USA	2009	Hospital-based	NI	NI	532	532	66.00±16.50	62.40±18.10	45.30	48.70
Hershow *et al* 68	USA	1990	Hospital-based	NI	NI	15	10	54.00	44.00	53.00	30.00
Hershow *et al* 68	USA	1990	Hospital-based	NI	NI	56	14	50.00	57.00	48.21	35.71
Hsu *et al* 69	USA	2001	Hospital-based	NI	NI	91	86	73.8±15.20	68±17.40	43.00	49.00
Isendahl *et al* 58	Sweden	2009	Hospital-based	Consecutive patients	Random	945	9390	65.62±15.7		58.90	
Jinnah *et al* 23	Bangladesh	NI	Hospital-based	Random selection	Random selection	150	150	NI	NI	NI	NI
Khurram *et al* 50	Pakistan	1998	Hospital-based	Consecutive patients	Consecutive patients	37	20	52.8±15.10	34.9±21.20	54.10	65.00
Kim *et al* 46	Korea	2008	Hospital-based	Consecutive patients	Consecutive patients	82	122	66.3±10.50	67.3±15.70	59.80	68.90
Manzur *et al* 56	Spain	1997	Hospital-based	Consecutive patients	Consecutive patients	50	98	69.06±13.90	58.33±18.24	45.00	65.30
Park *et al* 45	Korea	2007–2013	Hospital-based	Consecutive patients	Random	75	225	NI	NI	12.00	6.20
Ren *et al* 32	China	2015	Hospital-based	Consecutive patients	Not clear	98	49	NI	NI	NI	NI
Saade *et al* 70	USA	2000–2013	Hospital-based	NI	NI	428	59 041	62.95±12.10	66.75±5.76	100	100
Saibal *et al* 24	Bangladesh	2009	Hospital-based	Convenient and purposive	Convenient and purposive	47	43	56.3±12.20	35.7±10.50	68.10	72.10
Silva *et al* 27	Brazil	2002	Hospital-based	Consecutive patients	Consecutive patients	56	51	65.80	61.50		
Søraas *et al* 49	Norway	2010	Hospital-based	Consecutive patients	Random	100	190	55.00±19.00	64.00±17.00	12.00	12.00
Vinken *et al* 15	The Netherlands	2014	Hospital-based	Consecutive patients	NI	283	283	73.30±12.10	72.20±13.10	0.00	0.00
Wright *et al* 74	USA	1996	Hospital-based	Consecutive patients	Consecutive patients	118	317	NI	NI	30.50	15.10
Wright *et al* 75	USA	1996	Hospital-based	Consecutive patients	Consecutive patients	67	381	NI	NI	17.90	16.00

For an expanded version of this table (ie, containing more details about the study population in each original report and bye case and control groups) refer to online supplemental tables 4 and 7.

NI, no information available.

Evidence was mostly derived from cross-sectional (29 reports),^16 20–22 25 28 30 31 33–38 40 44 47 48 52 53 60–65 76 77^ and case–control (28 reports)^15 23 24 27 32 39 41–43 45 46 49 50 54–58 66–75 78^ studies; fewer followed a cohort (five reports)^26 29 51 59 61^ design (tables 1 and 2); of note one report contributed to both the cross-sectional and cohort count.^61^

Infection sites reported varied among urinary tract (34 reports),^15 20 23 26 28 30 33 36 38–41 43 45 47–49 53–55 57 62 63 65–67 69 70 74–78^ respiratory (11 reports),^21 24 28 31 32 37 46 64 68 69 78^ skin and soft tissue (6 reports),^22 34 52 59 60 62^ intra-abdominal (2 reports),^16 65^ surgical wounds (2 reports)^47 67^ and ophthalmic (1 report)^35^ (table 3).

**Table 3:** Adjusted association estimates of antibiotic-resistant from cross-sectional and cohort reports

Infection site	Author	Antibiotic	Measure of association	Adjustment
Urinary tract infection	Chen *et al* 76	Cefazolin	OR: 2.32 (1.32 to 4.07)	Sex
	Chiu *et al* 77	Cefazolin	OR: 4.17 (2.0 to 9.09)	Age 65 years; male gender; residents of healthcare facility; benign prostate hypertrophy; urinary tract infection within 1 years; NG tube; dysuria; frequency/urgency; temperature ≥38.3°C
	Ho *et al* 53	Amoxicilin-clavunate	OR: 2.54 (1.09 to 5.88)	Gender; genitourinary abnormalities; antibiotic given and susceptibility (vs no antibiotic): given amoxicillin-clavulanate; susceptible; given other antibiotic; susceptible
	Wu *et al* 30	Levofloxacin	OR: 3.80 (1.50 to 9.90)	Age; gender; recurrent urinary tract infection; prior hospitalisation in the past 6 months; prior antibiotic in the past 60 days; urinary function abnormality; indwelling urinary catheter; old stroke; altered consciousness; urinary symptoms; chills; fever; haematuria
Respiratory tract infection	Madaras-Kelly *et al* 64	Non-pseudomonal third generation cephalosporins (ceftriaxone or cefotaxime) or non-pseudomonal 8-methoxy fluoroquinolones (moxifloxacin; gatifloxacin); the VA preferred agents for treatment of community-acquired pneumonia	OR: 2.20 (1.20 to 4.30)	Nursing home residence or discharge ≤180 days prior to admission; positive methicillin-resistant *Staphylococcus aureus* status prior to admission; anti-pseudomonal fluoroquinolone exposure ≤365 days prior to admission; third generation cephalosporin exposure ≤365 days prior to admission; chronic inhaled corticosteroids
	Madaras-Kelly *et al* 64	Non-pseudomonal third generation cephalosporins (ceftriaxone or cefotaxime) or non-pseudomonal 8-methoxy fluoroquinolones (moxifloxacin; gatifloxacin); the Veterans Affairs preferred agents for treatment of CAP	OR: 1.70 (1.00 to 2.80)	Nursing home residence or discharge ≤180 days prior to admission; positive methicillin-resistant *Staphylococcus aureus* status prior to admission; cephalosporin exposure ≤365 days prior to admission; infusion therapy ≤30 days prior to admission; direct intense care unit admission on hospitalisation
	Micek *et al* 37	Aminoglycosides; antipseudomonal carbapenems; antipseudomonal cephalosporins; antipseudomonal fluoroquinolones; antipseudomonal penicillins plus β-lactamase inhibitors; monobactams; phosphonic acids and polymixins	OR: 1.90 (1.21 to 3.00)	Age; sex; residence in a community settings prior admission; residence in an inpatient rehabilitation facility prior to admission; antibiotics in the previous 30 days; COPD; solid tumour; dementia; intense care unit admission
Complicated skin and skin structure infections	Jääskeläinen *et al* 34	Carbapenem; piperacillin-tazobactam	OR: 1.67 (0.96 to 2.91)	Age; chronic renal failure; respiratory disease; injection drug abuse; abscess; cellulitis/fasciitis; number of days between symptoms start and diagnosis
	Jääskeläinen *et al* 34	Cefadroxil; cefotaxim; ceftriaxone; cefuroxime; cephalexin	OR: 1.07 (0.69 to 1.64)	Age; chronic renal failure; respiratory disease; injection drug abuse; abscess; cellulitis/fasciitis; number of days between symptoms start and diagnosis
	Jääskeläinen *et al* 34	Amoxicillin; benzylpenicillin; phenoxymethylpenicillin	OR: 0.94 (0.46 to 1.91)	Age; chronic renal failure; respiratory disease; injection drug abuse; abscess; cellulitis/fasciitis; number of days between symptoms start and diagnosis
	Jääskeläinen *et al* 34	Clindamycin; doxycyclin; fluoroquinolone; fusidic acid; linezolid; metronidazole; cotrimoxazole; tobramycin; vancomycin	OR: 0.79 (0.38 to 1.64)	Age; chronic renal failure; respiratory disease; injection drug abuse; abscess; cellulitis/fasciitis; number of days between symptoms start and diagnosis
	Jääskeläinen *et al* 34	Cloxacillin; flucloxacillin; other β-lactamase-stable penicillins	OR: 0.50 (0.24 to 1.08)	Age; chronic renal failure; respiratory disease; injection drug abuse; abscess; cellulitis/fasciitis; number of days between symptoms start and diagnosis

For an expanded version of this table (ie, containing more details about the bacteria included in each original report) refer to online supplemental table 8.

CAP, community-acquired pneumonia; COPD, chronic obstructive pulmonary disease.

### Infections

We report on adjusted association estimates as these were available in the original publications because these represent the most reliable and robust evidence (tables 3 and 4). Crude estimates and frequencies are available in online supplemental tables 3 and 4. Most studies reported that resistant infections were more frequent among people with T2DM in comparison to T2DM-free individuals; this observation was consistent across infection sites and study designs. Overall, regardless of the infection site, T2DM appears to be a risk factor for resistant infections (tables 3 and 4). Evidence to support this statement was weak only in a few studies as their estimates were not significant.^15 34 45^ Across infection sites, the comparison group was an infection with a non-resistant bacteria (rather than no infection or colonisation), except for two reports,^42 50^ where patients were colonised but did not develop an infection.

**Table 4:** Adjusted association estimates of antibiotic-resistant from case–control reports

Infection site	Author	Antibiotic	Bacteria	Measure of association	Adjustment
Urinary tract infection	Vinken *et al* 15	Nitrofurantoin; trimethoprim; fosfomycin; ciprofloxacin; amoxicillin/clavulanic acid and/or trimethoprim/sulfamethoxazol	*Escherichia coli*; *Enterococcus* spp; *Klebsiella pneumoniae*; *Proteus mirabilis*; *Klebsiella oxytoca*; *Pseudomonas aeruginosa*; and others 32 species	OR: 0.90 (0.40 to 1.80)	Age; hospital admission in preceding 12 months; elderly home resident; use of antibiotics in preceding 12 months; urinary tract infection in preceding 12 months; micturition complaints in preceding 6 weeks
	Colodner *et al* 43	Extended-spectrum beta-lactamases	*Escherichia coli* or *Klebsiella*	OR: 2.57 (1.20 to 5.51)	Age; *Escherichia coli* infection; infection; duration of antibiotic treatment; hospitalisation in the last 3 months; gender; age more than 60; underlying diseases (cardiovascular; gastrointestinal; genitourinary; recurrent urinary tract infection; neurological; malignancies)
	Wright *et al* 75	Trimethoprim-sulfamethoxazole	Coliform (*Escherichia coli*; *Proteus mirabilis*; *Klebsiella pneumoniae*; *Enterobacter species*; *Citrobacter freundi*; *Providencia* species and *Morganella morganii*)	OR: 3.10 (1.20 to 8.40)	Age; use of catheter; history of recurrent urinary tract infection; urological abnormality; neurological abnormality; recently in hospital; current antibiotic; current trimethoprim-sulfamethoxazole use
	Park *et al* 45	Amikacin; gentamicin; tobramycin; ciprofloxacin; levofloxacin; amoxicillun-clavulanate; piperacillin-tazobactam; trimethoprim-sulfamethoxazole; fluoroquinolones; aminoglycosides	*Escherichia coli*	OR: 1.69 (0.78 to 3.44)	Age >55; sex; urinary tract abnormalities; acute pyelonephritis recurrence and antibiotic use within the previous year
	Anesi *et al* 66	Extended-spectrum cephalosporin	Extended-spectrum cephalosporin-resistant *Enterobacteriaceae: Escherichia coli* (76%); *Klebsiella* species (13%) and *Enterobacter* species (9%)	OR: 2.91 (1.32 to 6.41)	Age; presentation to emergency department; trimethoprim sulfamethoxazole; receipt within prior 6 months
	Søraas *et al* 49	Extended-spectrum beta-lactamases (mecillinam; macrolides; tetracyclines; fluoroquinolones; nitrofurantoin; trimethoprim or trimethoprim/sulfamethoxazole; b-lactams except mecillinam; methenamine hippurate	*Escherichia coli* or *Klebsiella*	OR: 3.20 (1.00 to 11.00)	Travel destinations; recreational swimming past year; fish meals per week; dinner at restaurant >2/month; close occupational contact with humans; bath or shower <2/week; digestive problems
Respiratory tract infection	Kim *et al* 46	Carbapenems (meropenem; imipenem)	Gram-negative bacteria *or Stenotrophomonas maltophili*	OR: 2.82 (1.25 to 6.38)	Neurological disease; Macabe and Jackson classification; ventilator-associated pneumonia; APACHE II; radiological score; prior antibiotic usage

The association estimates from cross-sectional or cohort studies were as high as fivefold (OR=5.2, 95% CI 1.4 to 19.8) for surgical wounds, blood, urinary and respiratory tract or burn infections;^47^ other studies also reported a similar association estimate for urinary tract, skin or soft tissue infections (OR=5.1, 95% CI 2.1 to 18.6).^62^ On the other hand, the smallest estimate was 1.70 (95% CI 1.0 to 2.8) for community-acquired pneumonia.^64^

The association estimates from case–control studies showed a similar pattern. The largest estimate showed an OR of 6.4 (95% CI 2.1 to 19.3) for respiratory, urinary, wound or bloodstream infections.^69^ At the other extreme, people with T2DM had 50% higher odds of a resistant urinary or bloodstream infection (OR=1.5, 95% CI 1.2 to 1.8).^70^

### Meta-analysis

We further elaborated on urinary tract infections because most studies addressed this condition. The pooled OR across 10 reports,^15 30 43 45 49 53 66 75–77^ supported the premise that people with T2DM were most likely to have a resistant infection in the urinary tract, rather than a non-resistant infection: OR=2.42 (95% CI 1.83 to 3.20; I^2^ 19.1%; 3675 subjects). When only cross-sectional studies were pooled (all hospital-based), the summary estimate based on these four studies was^30 53 76 77^: OR=2.92 (95% CI 2.02 to 4.21; I^2^ 00.0%; 1312 subjects); alternatively, when only case–control studies were pooled (all hospital-based), the summary estimate based on these six studies was^15 43 45 49 66 75^: OR=2.07 (95% CI 1.37 to 3.12; I^2^ 30.3%; 2363 subjects). These reports analysed a range of bacteria and antibiotics, and consistently suggested that people with T2DM were most likely to experience a resistant infection in the urinary tract (tables 3 and 4), rather than a non-resistant infection.

There were also at least four estimates to conduct a meta-analysis for respiratory tract infections.^32 37 64^ This analysis also suggested that people with T2DM showed higher odds of resistant infections: OR=2.35 (95% CI 1.49 to 3.69; I^2^ 58.1%; 1637 subjects). It is noteworthy that one study^64^ contributed with two estimates from the same study population (375 subjects), thus it was considered twice for this meta-analysis. There were three cross-sectional hospital-based studies,^37 64^ and one case–control hospital-based study^32^; therefore, further stratification by study design was not possible. These reports analysed a range of bacteria and antibiotics, consistently suggesting that resistant infections were more likely in people with T2DM (tables 3 and 4). Overall, people with T2DM appear to be at higher risk of a resistant respiratory infection.

Five estimates from the same study informed the pooled analysis for complicated skin infections: OR=0.98 (95% CI 0.68 to 1.41; I^2^ 43.3%; 390 subjects). This was a cross-sectional hospital-based study, which analysed different antibiotics and bacteria (table 3).^34^ The available evidence is still inconclusive on whether people with T2DM have higher risk of complicated skin infections. Similarly, for other infection sites, it was not possible to reach strong conclusions or to conduct a meta-analysis, because the body of evidence was small or there was large heterogeneity (tables 3 and 4).

### Risk of bias

On average, the summarised reports had 5.9 stars in the risk of bias assessment tool (online supplemental table 5), with just a few showing fewer than four stars mostly because some criteria did not apply for the study design, or information was not available or was unclear.^20 28 38 48 52^

## Discussion

### Summary of the evidence

The evidence suggests that, in comparison to T2DM-free subjects, people with T2DM who acquire a urinary tract and respiratory infection, are more likely to experience a resistant infection. The evidence for other infection sites was less conclusive because of fewer reports and large heterogeneity in the outcomes. The body of evidence studying T2DM as an associated factor for resistant infections has increased, suggesting that researchers and practitioners find this topic relevant. This work has summarised and pooled available evidence and delivered strong conclusions about two infection sites, while signalling other infections sites that warrant further research.

### Pathways behind T2DM and resistant infections

A comprehensive discussion on the immunological or pharmacological mechanisms involved in the association between T2DM and resistant infections is beyond the scope of this work. Nonetheless, we acknowledge that T2DM negatively interacts with the immune system and could be a risk factor for infections.^79–83^ In this line, other conditions related with T2DM, obesity for example, have been associated with an increased risk of infections due to the role of adipose tissue in the production of proinflammatory cytokines (tumor necrosis factor (TNF)-α, interleukin (IL) 6, IL-1β, IL-18, monocyte chemoattractant protein (MCP)-1), proinflammatory adipokines and other inflammatory products;^84 85^ another point is the pharmacokinetics of antibiotics in obese population that can lead to suboptimal levels of antibiotic concentrations and increase the risk for antibiotic resistance.^86^

Whether the impaired immune system is responsible for higher risk of resistant infections in people with T2DM, has not been studied to the best of our knowledge. Another pending question is whether the frequency with which people with T2DM visit clinics or hospitals is a risk factor for resistant infections. That is, people with T2DM have more contact with healthcare facilities because of regular control visits or other related complications. In these visits, they could acquire infections with in-hospital bacteria, perhaps more likely to be resistant.

### Public health and clinical practice implications

The estimates herein summarised could inform clinical practice for people with T2DM. Our estimates support the fact that T2DM are more likely to have resistant infections, particularly urinary tract and respiratory infections. Empirical treatment for these infection sites in people with T2DM needs to be carefully thought; that is, the empirical treatment for the general population may not be the best option in people with T2DM. This does not imply starting treatment with a very powerful antibiotic, but to carefully consider available options and if possible, request an antibiotic sensitivity test to inform the empirical treatment choice.

From a health economics perspective, treatment failure with a first-line antibiotic because of antibiotic resistance in people with T2DM would impose a large economic burden.^10 87^ These T2DM patients may need a second appointment with their medic, and start a different course of antibiotics; in the worst-case scenario, the infection could progress and develop some complication. These are additional costs for the health system or the patient.

Resistant infections are a major concern in infectious diseases medicine. For example, the burden of resistant tuberculosis^88^ has received great attention, it is frequently monitored, and guides diagnosis and treatment allocation. Talking about resistant infections in the field of non-communicable diseases is new, yet some authors have already highlighted the links between communicable and non-communicable diseases.^5–8^ A surveillance system of antibiotic resistance profiles among people with T2DM could be implemented to identify the most dangerous bacteria, select the best treatment considering other concomitant risk factor such as obesity,^86^ and monitor trends of the resistance patterns in the T2DM community. An antibiotic resistance surveillance programme could inform local and international guidelines for infections in people with T2DM.

There are clinical practice guidelines for diabetic food infections,^89^ which represent a great burden on T2DM patients. Guidelines for other infections in people with T2DM are less common. Although available guidelines, for example, those for urinary tract infections,^90 91^ acknowledge T2DM as a risk factor for asymptomatic bacteriuria or complicated infections, little is discussed about antibiotic resistance or tailored treatment choices for people with T2DM. Our work, accounting for its limitations, could be adopted by these guidelines to suggest some pragmatic approaches for people with T2DM. For example: (i) carefully contemplate the empirical treatment considering that the choices for the general population may not be ideal for T2DM patients or (ii) consider an antibiogram before starting any empirical treatment. Ideally, experimental studies would come to further strengthen—or reject—these suggestions for the benefit of T2DM patients. We advocate for a map of antibiotic-resistant profile in people with T2DM, at least for the urinary tract and respiratory infections, and diabetic foot infections.^89^ This evidence would have a positive impact on guiding empirical treatment for people with T2DM.

It is worth noting that most of the original studies herein summarised were hospital-based or conducted with captive populations (eg, nursing homes). Whether the same findings would apply to the general population in community-based or population-based studies with implications in primary care, deserves further investigation.

### Limitations

We conducted a comprehensive review following standard methods. However, there are also some limitations to acknowledge. First, it was not always specified whether the original studies referred to T2DM patients alone; that is, we cannot be certain that type 1 diabetes mellitus patients were fully excluded. However, because we focused on adults, in whom the overall prevalence of T2DM is the largest relative to other types of diabetes, it is reasonable to consider that only (or mostly) T2DM patients were studied. Second, in many studies the sample size was limited particularly when authors tried to look at specific subgroups. More comprehensive and larger research is needed in this field, particularly with other infection sites where evidence is much limited. Third, a consequence of a limited sample size is the lack of multivariable models. It was challenging to ascertain whether T2DM is an independent risk factor for resistant infections. Metabolic control as per HbA1c levels and hyperglycaemic status are relevant variables to account for.^79–83^ Future studies should include HbA1c and other variables. Fourth, original studies followed different designs and sampling frameworks. Electronic health records could provide a remarkable and timely opportunity to further explore the role of T2DM in resistant infections.

## Conclusions

This systematic review and meta-analysis found evidence signalling that people with T2DM are more likely to experience resistant urinary tract and respiratory infections. Although the evidence for other infection sites was less conclusive, it already pinpoints that people with T2DM are more likely to have resistant infections regardless of the infection site. This evidence, along with clinical knowledge and decision-sharing, could guide empirical treatment for patients with T2DM and infections.

**What is already known on this subject**

- There is a growing body of evidence about diabetes as a risk factor for infectious diseases and antibiotic-resistant infections; however, there is great heterogeneity among individual reports and most studies included small samples. This sparse evidence limits our understanding of diabetes as a compelling risk factor for antibiotic-resistant infections.

**What this study adds**

- Diabetes appears to be a strong risk factor for antibiotic-resistant infections, particularly urinary tract and respiratory infections. Although evidence for other infection sites was limited, in terms of quantity, quality and with great heterogeneity, their findings already suggested that diabetes was associated with higher risk of resistant infections. This evidence could inform clinical guidelines for infectious diseases, with focus on people with diabetes, which is a large and growing proportion of the general population.
