# Supplementary material for: Type 2 diabetes mellitus and antibiotic-resistant infections: a systematic review and meta-analysis
Source: J Epidemiol Community Health. 2021 Jul 29;76(1):75–84. doi: 10.1136/jech-2020-216029 (PMC8666814; doi:10.1136/jech-2020-216029)
Supplement: Supplementary data [file jech-2020-216029supp001.pdf]

Type 2 diabetes mellitus and antibiotic-resistant infections: A systematic review and meta-analysis

Correspondence author:

Rodrigo M Carrillo-Larco, MD

Department of Epidemiology and Biostatistics

School of Public Health

Imperial College London

rcarrill@ic.ac.uk

Supplementary Table 1: PRISMA checklist....3

Supplementary Table 2: Search terms....5

Supplementary Table 3: Cross-sectional and cohort studies....6

Supplementary Table 4: Case-control studies....21

Supplementary Table 5: Risk of bias....33

Supplementary Table 6: Characteristics of cross-sectional and cohort reports (expanded from Table 1 in the main paper)....35

Supplementary Table 7: Characteristics of case-control reports (expanded from Table 2 in the main paper)....40

Supplementary Table 8: Adjusted association estimates of antibiotic-resistant from cross-sectional and cohort reports (expanded from Table 3 in the main paper)....46

Carrillo-Larco RM, *et al. J Epidemiol Community Health* 2021; 76:75–84. doi: 10.1136/jech-2020-216029

Supplementary Table 1: PRISMA checklist

Section/topic	#	Checklist item	Reported on page #
**TITLE**			
Title	1	Identify the report as a systematic review, meta-analysis, or both.	01
**ABSTRACT**			
Structured summary	2	Provide a structured summary including, as applicable: background; objectives; data sources; study eligibility criteria, participants, and interventions; study appraisal and synthesis methods; results; limitations; conclusions and implications of key findings; systematic review registration number.	03
**INTRODUCTION**			
Rationale	3	Describe the rationale for the review in the context of what is already known.	04
Objectives	4	Provide an explicit statement of questions being addressed with reference to participants, interventions, comparisons, outcomes, and study design (PICOS).	04
**METHODS**			
Protocol and registration	5	Indicate if a review protocol exists, if and where it can be accessed (e.g., Web address), and, if available, provide registration information including registration number.	04-05
Eligibility criteria	6	Specify study characteristics (e.g., PICOS, length of follow-up) and report characteristics (e.g., years considered, language, publication status) used as criteria for eligibility, giving rationale.	05
Information sources	7	Describe all information sources (e.g., databases with dates of coverage, contact with study authors to identify additional studies) in the search and date last searched.	05
Search	8	Present full electronic search strategy for at least one database, including any limits used, such that it could be repeated.	05
Study selection	9	State the process for selecting studies (i.e., screening, eligibility, included in systematic review, and, if applicable, included in the meta-analysis).	05
Data collection process	10	Describe method of data extraction from reports (e.g., piloted forms, independently, in duplicate) and any processes for obtaining and confirming data from investigators.	06
Data items	11	List and define all variables for which data were sought (e.g., PICOS, funding sources) and any assumptions and simplifications made.	06
Risk of bias in individual studies	12	Describe methods used for assessing risk of bias of individual studies (including specification of whether this was done at the study or outcome level), and how this information is to be used in any data synthesis.	06
Summary measures	13	State the principal summary measures (e.g., risk ratio, difference in means).	06-07
Synthesis of results	14	Describe the methods of handling data and combining results of studies, if done, including measures of consistency (e.g., I^2) for each meta-analysis.	06-07
Risk of bias across studies	15	Specify any assessment of risk of bias that may affect the cumulative evidence (e.g., publication bias, selective reporting within studies).	NA
Additional analyses	16	Describe methods of additional analyses (e.g., sensitivity or subgroup analyses, meta-regression), if done, indicating which were pre-specified.	NA
**RESULTS**			
Study selection	17	Give numbers of studies screened, assessed for eligibility, and included in the review, with reasons for exclusions at each stage, ideally with a flow diagram.	07

Study characteristics	18	For each study, present characteristics for which data were extracted (e.g., study size, PICOS, follow-up period) and provide the citations.	07
Risk of bias within studies	19	Present data on risk of bias of each study and, if available, any outcome level assessment (see item 12).	09
Results of individual studies	20	For all outcomes considered (benefits or harms), present, for each study: (a) simple summary data for each intervention group (b) effect estimates and confidence intervals, ideally with a forest plot.	08
Synthesis of results	21	Present results of each meta-analysis done, including confidence intervals and measures of consistency.	08
Risk of bias across studies	22	Present results of any assessment of risk of bias across studies (see Item 15).	NA
Additional analysis	23	Give results of additional analyses, if done (e.g., sensitivity or subgroup analyses, meta-regression [see Item 16]).	NA
**DISCUSSION**			
Summary of evidence	24	Summarize the main findings including the strength of evidence for each main outcome; consider their relevance to key groups (e.g., healthcare providers, users, and policy makers).	10
Limitations	25	Discuss limitations at study and outcome level (e.g., risk of bias), and at review-level (e.g., incomplete retrieval of identified research, reporting bias).	12
Conclusions	26	Provide a general interpretation of the results in the context of other evidence, and implications for future research.	14
**FUNDING**			
Funding	27	Describe sources of funding for the systematic review and other support (e.g., supply of data); role of funders for the systematic review.	02

Supplementary Table 2: Search terms

1	diabet*.mp.
2	exp Diabetes Mellitus/
3	type 2 diabetes.mp.
4	exp Diabetes Mellitus, Type 2/
5	T2D*.mp.
6	1 or 2 … or 5
7	antimicrobial resistance.mp.
8	antibiotic resistance.mp.
9	drug resistance.mp.
10	drug susceptibility.mp.
11	anti-bacterial resistance.mp.
12	exp Drug Resistance, Bacterial/
13	exp Drug Resistance, Microbial/
14	exp Microbial Sensitivity Tests/
15	multidrug resistance.mp.
16	7 or 8 … or 15
17	6 and 16
18	exp animals/ not humans.sh.
19	17 not 18

Supplementary Table 3: Cross-sectional and cohort studies

Año y autor	Infection site	Antibiotic	Bacteria	Diabetes type	Diabetics with ant. resistance	Diabetics without ant. resistance	Non-diabetics with ant. resistance	Non-diabetics without ant. resistance	Measure of association	Adjustment
Bailey et al. 2013	Urinary tract infection	SMX-TMP	*Escherichia coli*	Diabetes mellitus	13	28	48	133	PR: 1.20 [0.72 ; 1.99]	NA
Bailey et al. 2013	Urinary tract infection	Levofloxacin	*Escherichia coli*	Diabetes mellitus	10	31	29	152	PR: 1.52 [0.81 ; 2.87]	NA
Baillargeon et al. 2004	In general (Skin or soft tissue or body fluid)	NI	*Methicillin resistant Staphylococcus aureus*	Type 1 and 2 Diabetes mellitus	296	NI	NI	NI	RR: 1.70 [1.50 ; 1.90]	NA
Baillargeon et al. 2004	In general (Skin or soft tissue or body fluid)	NI	*Methicillin resistant Staphylococcus aureus*	Type 1 and 2 Diabetes mellitus	296	NI	NI	NI	HR: 1.90 [1.70 ; 2.20]	NA
Benenson et al. 2005	Bacteriemia (Urinary tract infection and other)	Gentamicin	*Gram negative bacteria (Escherichia coli, Klebsiella pneumoniae, Proteus mirabilis, others)*	Diabetes mellitus	NI	NI	NI	NI	OR: 0.30 [0.10 ; 1.30]	Not clear
Benenson et al. 2005	Bacteriemia (Urinary tract infection and other)	Ciprofloxacin	*Gram negative bacteria (Escherichia coli, Klebsiella pneumoniae, Proteus mirabilis, others)*	Diabetes mellitus	NI	NI	NI	NI	OR: 0.50 [0.20 ; 1.60]	Not clear
Bonadio et al. 2006	Urinary tract infection	Ampicilin	*Escherichia coli*	Type 1 and 2 Diabetes mellitus	46	111	147	343	PR: 0.98 [0.74 ; 1.29]	NA
Bonadio et al. 2006	Urinary tract infection	Cotrimoxazol	*Escherichia coli*	Type 1 and 2 Diabetes mellitus	29	122	77	364	PR: 1.10 [0.75 ; 1.62]	NA
Bonadio et al. 2006	Urinary tract infection	Ciprofloxacin	*Escherichia coli*	Type 1 and 2 Diabetes mellitus	18	136	31	432	PR: 1.23 [0.68 ; 2.20]	NA

Bonadio et al. 2006	Urinary tract infection	Nitrofurantoin	*Escherichia coli*	Type 1 and 2 Diabetes mellitus	15	163	34	461	PR: 1.75 [1.01 ; 3.03]	NA
Chen et al. 2013	Urinary tract infection	Cefazolin	*Escherichia coli (69%), K. pneumoniae (7%), Pseudomonas aeruginosa (5%), Proteus mirabilis (4%), Enterobacter (3%) and Citrobacter species (2%).*	Diabetes mellitus	65	87	77	191	OR: 2.32 [1.32 ; 4.07]	Sex (ref. female)
Chiquet et al. 2015	Ophtalmic infection	Methicillin	*Coagulase-negative staphylococci: Staphylococcus epidermidis (56 strains, 82.4%), Staphylococcus lugdunensis (n = 5), S. warneri (n = 3), Staphylococcus hominis (n = 1), Staphylococcus haemolyticus (n = 2) and Staphylococcus saprophyticus (n = 1).*	Diabetes mellitus	9.96	2	4	52	PR: 11.66 [4.38 ; 31.00]	NA
Chiu et al. 2017	Urinary tract infection	Cefazolin	*Escherichia coli (54.5%) was most commonly found. Other common uropathogens were Klebsiella pneumoniae (13.1%), Enterococcus spp. (7.1%), Pseudomonas aeruginosa (4.6%), and Proteus mirabilis (3.5%). Of 115 UTI-originated bacteremic episodes, 16*	Diabetes mellitus	60	53	51	106	OR: 4.17 [2.0 ; 9.09]	Age 65 y, Male gender, Residents of health care facility, bening prostate hyperthrophy, UTI within 1 y, NG tube, Dysuria, frequency/urgency, Temperature >= 38.3 C
Chong et al. 2013	Hand infection	NI	*Methicillin sensitive Staphylococcus aureus*	Diabetes mellitus	2	7	3	26	PR: 2.15 [0.42 ; 10.91]	NA
Chong et al. 2013	Hand infection	NI	*Methicillin sensitive Staphylococcus aureus*	Diabetes mellitus	1	11	1	40	PR: 3.42 [0.23 ; 50.65]	NA

Ho et al. 2019	Urinary tract infection	Amoxicilin-clavunate	*Escherichia coli, Klebsiella sp., Proteus mirabilis, Enterobacteriaceae, or Staphylococcus saprophyticus only*	Diabetes mellitus	NI	NI	NI	NI	OR: 2.54 [1.09 ; 5.88]	Gender, Genitourinary abnormalities, Antibiotic given and susceptibility (vs no antibiotic) (Given amoxicillin-clavulanate, susceptible, Given other antibiotic, susceptible)
Jaaskelainen et al. 2017	Complicated Skin and Skin Structure Infections	Cefadroxil; cefotaxim; ceftriaxone; cefuroxime; cephalexin.	*Methicillin-sensitive Staphylococcus aureus; Streptococcus pyogenes; Streptococcus agalactiae; β-hemolytic streptococci; Streptococcus pneumoniae; α-hemolytic streptococci; Enterobacteriacae; Pseudomonas; Anaerobic bacteria; Enterococi; others*	Diabetes mellitus	60	58	133	137	OR: 0.50 [0.24 ; 1.08]	Age; Chronic renal failure; respitarory disease; Injection drug abuse; Abscess; Cellulitis/fascitis; Number of Days Between Symptoms Start and Diagnosis
Jaaskelainen et al. 2017	Complicated Skin and Skin Structure Infections	Carbapenem; piperacillin-tazobactam	*Methicillin-sensitive Staphylococcus aureus; Streptococcus pyogenes; Streptococcus agalactiae; β-hemolytic streptococci; Streptococcus pneumoniae; α-hemolytic streptococci; Enterobacteriacae; Pseudomonas; Anaerobic bacteria; Enterococi; others*	Diabetes mellitus	26	92	39	231	OR: 0.79 [0.38 ; 1.64]	Age; Chronic renal failure; respitarory disease; Injection drug abuse; Abscess; Cellulitis/fascitis; Number of Days Between Symptoms Start and Diagnosis
Jaaskelainen et al. 2017	Complicated Skin and Skin Structure Infections	Amoxicillin; benzylpenicillin; phenoxymethylpenicillin.	*Methicillin-sensitive Staphylococcus aureus; Streptococcus pyogenes; Streptococcus agalactiae; β-hemolytic streptococci; Streptococcus pneumoniae; α-hemolytic streptococci; Enterobacteriacae; Pseudomonas; Anaerobic bacteria; Enterococi; others*	Diabetes mellitus	12	106	29	241	OR: 0.94 [0.46 ; 1.91]	Age; Chronic renal failure; respitarory disease; Injection drug abuse; Abscess; Cellulitis/fascitis; Number of Days Between Symptoms Start and Diagnosis

Carrillo-Larco RM, *et al. J Epidemiol Community Health* 2021; 76:75–84. doi: 10.1136/jech-2020-216029

Jaaskelainen et al. 2017	Complicated Skin and Skin Structure Infections	Clindamycin; doxycyclin; fluoroquinolone; fusidic acid; linezolid; metronidazole; cotrimoxazole; tobramycin; vancomycin.	*Methicillin-sensitive Staphylococcus aureus; Streptococcus pyogenes; Streptococcus agalactiae; β-hemolytic streptococci; Streptococcus pneumoniae; α-hemolytic streptococci; Enterobacteriacae; Pseudomonas; Anaerobic bacteria; Enterococi; others*	Diabetes mellitus	11	107	31	239	OR: 1.07 [0.69 ; 1.64]	Age; Chronic renal failure; respitarory disease; Injection drug abuse; Abscess; Cellulitis/fascitis; Number of Days Between Symptoms Start and Diagnosis
Jaaskelainen et al. 2017	Complicated Skin and Skin Structure Infections	Cloxacillin; flucloxacillin; other β-lactamase-stable penicillins	*Methicillin-sensitive Staphylococcus aureus; Streptococcus pyogenes; Streptococcus agalactiae; β-hemolytic streptococci; Streptococcus pneumoniae; α-hemolytic streptococci; Enterobacteriacae; Pseudomonas; Anaerobic bacteria; Enterococi; others*	Diabetes mellitus	9	109	38	232	PR: 1.43 [0.13 ; 15.10]	NA
Jaaskelainen et al. 2017	Complicated Skin and Skin Structure Infections	Methicillin Resistant Staphylococcus aureus	*Staphylococcus aureus*	Diabetes mellitus	1	26	2	75	OR: 1.67 [0.96 ; 2.91]	Age; Chronic renal failure; respitarory disease; Injection drug abuse; Abscess; Cellulitis/fascitis; Number of Days Between Symptoms Start and Diagnosis
Kistler et al. 2019	Hand infection	Levofloxcin	*Methicillin-Resistant Staphylococcus aureus*	Diabetes mellitus	NI	NI	NI	NI	OR: 0.58	NA
Kistler et al. 2019	Hand infection	Clindamycin	*Methicillin-Resistant Staphylococcus aureus*	Diabetes mellitus	NI	NI	NI	NI	OR: 2.48	NA

Kurup et al. 2019	Skin (Ulcer) infection	Piperacillin, Septrin, Erythromycin, Clindamycin, Chloramphenicol, Ciprofloxacin, Linezolid, Amikacin, Ampicillin, Cefazolin, Vancomycin, Imipenem, Piperacillin, Cefotaxime, Ceftazidime, Ceftriaxone, Cefuroxime, Ertapenem, Erythromycin, Gentamycin, Tobramycin	*Gram negative (Acinetobacter sp., Citrobacter sp., Enterobacter sp., Escherichia coli, P. aeruginosa, K. pneumoniae, M. morganni, Proteus sp., Providencia sp., others) and Gram positive bacteria (Enterococcus sp, MRSA, Streptococcus sp., MSSA)*	Type 2 Diabetes mellitus	32	48	25	56	PR: 1.03 [0.52 ; 2.06]	NA
Kurup et al. 2019	Skin (Ulcer) infection	Piperacillin, Septrin, Erythromycin, Clindamycin, Chloramphenicol, Ciprofloxacin, Linezolid, Amikacin, Ampicillin, Cefazolin, Vancomycin, Imipenem, Piperacillin, Cefotaxime, Ceftazidime, Ceftriaxone, Cefuroxime, Ertapenem, Erythromycin, Gentamycin, Tobramycin	*Gram negative (Acinetobacter sp., Citrobacter sp., Enterobacter sp., Escherichia coli, P. aeruginosa, K. pneumoniae, M. morganni, Proteus sp., Providencia sp., others) and Gram positive bacteria (Enterococcus sp, MRSA, Streptococcus sp., MSSA)*	Type 1 Diabetes mellitus	7	15	25	56	PR: 1.30 [0.85 ; 1.98]	NA
Laupland et al. 2008	Bacteriemia (Bone and joint, soft tissue, respiratory, endovascular, intra-abdominal/pelvic, CNS, other)	Methicillin Resistant Staphylococcus aureus	*Staphylococcus aureus*	Diabetes mellitus	NI	NI	NI	NI	RR: 14.70 [10.4 ; 20.6]	Not clear

Levin et al. 2010	Nosocomial Respiratory tract infection , bacteremia (primary and secondary), and urinary tract infections	Third generation cephalosporin, fluoroquinolone, carbepenem, vancomycin, methicillin	*Acinetobacter baumannii, Enterobacter cloacae , Escherichia coli , Klebsiella pneumoniae , Pseudomonas aeruginosa, Enterococcus faecium , Staphylococcus aureus*	Diabetes mellitus	21	52	61	289	PR: 1.65 [1.08 ; 2.53]	NA
Libert et al. 2008	Bacteriemia (intravascular device, primary BSI, cutaneous, osteo-articular, respiratory, decubitus ulcers, postoperative wound, urinary, other)	Methicillin Resistant Staphylococcus aureus	*Staphylococcus aureus*	Diabetes mellitus	22	22	44	66	OR: 3.83 [1.16 ; 12.66]	Not clear (Stepwise multinomial logistic regression was used for multivariate analysis.)
Liu et al. 2019	Respiratory tract infection	Ampicilin	*Klebsiella pneumoniae*	Diabetes mellitus	146	7	269	16	PR: 0.70 [0.50 ; 0.98]	NA
Liu et al. 2019	Respiratory tract infection	Cefazolin	*Klebsiella pneumoniae*	Diabetes mellitus	85	65	160	121	PR: 0.71 [0.51 ; 1.00]	NA
Liu et al. 2019	Respiratory tract infection	Ceftriaxone	*Klebsiella pneumoniae*	Diabetes mellitus	71	81	137	145	PR: 0.81 [0.57 ; 1.16]	NA
Liu et al. 2019	Respiratory tract infection	Unasyn	*Klebsiella pneumoniae*	Diabetes mellitus	61	92	120	164	PR: 0.85 [0.65 ; 1.11]	NA
Liu et al. 2019	Respiratory tract infection	Aztreonam	*Klebsiella pneumoniae*	Diabetes mellitus	59	92	123	158	PR: 0.86 [0.63 ; 1.17]	NA
Liu et al. 2019	Respiratory tract infection	Nitrofurantion	*Klebsiella pneumoniae*	Diabetes mellitus	59	88	107	159	PR: 0.86 [0.64 ; 1.17]	NA
Liu et al. 2019	Respiratory tract infection	Ciprofloxacin	*Klebsiella pneumoniae*	Diabetes mellitus	58	96	104	181	PR: 0.88 [0.62 ; 1.23]	NA

Liu et al. 2019	Respiratory tract infection	Ceftazidime	*Klebsiella pneumoniae*	Diabetes mellitus	57	97	109	176	PR: 0.88 [0.66 ; 1.17]	NA
Liu et al. 2019	Respiratory tract infection	Gentamycin	*Klebsiella pneumoniae*	Diabetes mellitus	51	103	111	175	PR: 0.88 [0.63 ; 1.23]	NA
Liu et al. 2019	Respiratory tract infection	Ertapenem	*Klebsiella pneumoniae*	Diabetes mellitus	49	100	101	179	PR: 0.89 [0.70 ; 1.13]	NA
Liu et al. 2019	Respiratory tract infection	Cefepime	*Klebsiella pneumoniae*	Diabetes mellitus	48	106	92	194	PR: 0.91 [0.69 ; 1.20]	NA
Liu et al. 2019	Respiratory tract infection	Imipenem	*Klebsiella pneumoniae*	Diabetes mellitus	48	106	92	194	PR: 0.92 [0.70 ; 1.23]	NA
Liu et al. 2019	Respiratory tract infection	Levofloxcin	*Klebsiella pneumoniae*	Diabetes mellitus	48	103	97	185	PR: 0.94 [0.74 ; 1.20]	NA
Liu et al. 2019	Respiratory tract infection	Cefotetan	*Klebsiella pneumoniae*	Diabetes mellitus	47	104	100	182	PR: 0.94 [0.69 ; 1.29]	NA
Liu et al. 2019	Respiratory tract infection	PTZ	*Klebsiella pneumoniae*	Diabetes mellitus	43	111	84	200	PR: 0.96 [0.78 ; 1.18]	NA
Liu et al. 2019	Respiratory tract infection	Tobramycin	*Klebsiella pneumoniae*	Diabetes mellitus	43	108	92	186	PR: 0.97 [0.75 ; 1.25]	NA
Liu et al. 2019	Respiratory tract infection	Amilkacin	*Klebsiella pneumoniae*	Diabetes mellitus	38	116	80	206	PR: 0.97 [0.73 ; 1.29]	NA
Liu et al. 2019	Respiratory tract infection	CRKP	*Klebsiella pneumoniae*	Diabetes mellitus	37	77	92	151	PR: 0.97 [0.73 ; 1.29]	NA
Liu et al. 2019	Respiratory tract infection	SMZ	*Klebsiella pneumoniae*	Diabetes mellitus	35	119	93	193	PR: 1.00 [0.84 ; 1.18]	NA
Liu et al. 2019	Respiratory tract infection	Sulperaxone	*Klebsiella pneumoniae*	Diabetes mellitus	34	96	81	190	PR: 1.00 [0.78 ; 1.28]	NA
Liu et al. 2019	Respiratory tract infection	Fosfomycin	*Klebsiella pneumoniae*	Diabetes mellitus	31	85	97	161	PR: 1.01 [0.97 ; 1.06]	NA
Liu et al. 2019	Respiratory tract infection	Meropenem	*Klebsiella pneumoniae*	Diabetes mellitus	30	89	77	171	PR: 1.03 [0.80 ; 1.33]	NA
Liu et al. 2019	Respiratory tract infection	ESBL-KP	*Klebsiella pneumoniae*	Diabetes mellitus	27	120	48	222	PR: 1.03 [0.67 ; 1.58]	NA

Liu et al. 2019	Respiratory tract infection	Tigecyclin	*Klebsiella pneumoniae*	Diabetes mellitus	5	99	10	211	PR: 1.06 [0.37 ; 3.03]	NA
Lye et al. 1992	Urinary tract infection	Cephalexin	*All organisms*	Diabetes mellitus	90	41	162	55	PR: 0.60 [0.40 ; 0.90]	NA
Lye et al. 1992	Urinary tract infection	Getamicin	*All organisms*	Diabetes mellitus	88	43	174	43	PR: 0.73 [0.41 ; 1.29]	NA
Lye et al. 1992	Urinary tract infection	SMX-TMP	*All organisms*	Diabetes mellitus	78	53	141	76	PR: 0.76 [0.57 ; 1.02]	NA
Lye et al. 1992	Urinary tract infection	Getamicin	*All organisms*	Diabetes mellitus	67	108	27	28	PR: 0.78 [0.56 ; 1.08]	NA
Lye et al. 1992	Urinary tract infection	Getamicin	*Escherichia coli*	Diabetes mellitus	56	6	136	12	PR: 0.79 [0.51 ; 1.23]	NA
Lye et al. 1992	Urinary tract infection	Ampicilin	*All organisms*	Diabetes mellitus	49	82	101	116	PR: 0.80 [0.62 ; 1.05]	NA
Lye et al. 1992	Urinary tract infection	SMX-TMP	*All organisms*	Diabetes mellitus	48	127	19	36	PR: 0.84 [0.73 ; 0.96]	NA
Lye et al. 1992	Urinary tract infection	Cephalexin	*Escherichia coli*	Diabetes mellitus	47	15	129	19	PR: 0.85 [0.67 ; 1.08]	NA
Lye et al. 1992	Urinary tract infection	Cephalexin	*All organisms*	Diabetes mellitus	44	131	23	32	PR: 0.87 [0.75 ; 1.01]	NA
Lye et al. 1992	Urinary tract infection	SMX-TMP	*Escherichia coli*	Diabetes mellitus	36	26	101	47	PR: 0.92 [0.77 ; 1.09]	NA
Lye et al. 1992	Urinary tract infection	Ampicilin	*Escherichia coli*	Diabetes mellitus	31	31	79	69	PR: 0.92 [0.8 ; 1.06]	NA
Lye et al. 1992	Urinary tract infection	Ampicilin	*All organisms*	Diabetes mellitus	30	145	13	42	PR: 0.94 [0.7 ; 1.25]	NA
Lye et al. 1992	Urinary tract infection	Cephalexin	*Klebsiella spp*	Diabetes mellitus	26	40	0	0	PR: 0.98 [0.89 ; 1.08]	NA
Lye et al. 1992	Urinary tract infection	Cephalexin	*Klebsiella spp*	Diabetes mellitus	26	9	14	8	PR: 1.17 [0.81 ; 1.69]	NA
Lye et al. 1992	Urinary tract infection	Getamicin	*Escherichia coli*	Diabetes mellitus	26	6	0	0	PR: 1.2 [0.77 ; 1.89]	NA

Lye et al. 1992	Urinary tract infection	Getamicin	*Klebsiella spp*	Diabetes mellitus	25	41	0	0	PR: 1.26 [0.12 ; 13.06]	NA
Lye et al. 1992	Urinary tract infection	SMX-TMP	*Klebsiella spp*	Diabetes mellitus	23	12	12	10		NA
Lye et al. 1992	Urinary tract infection	Getamicin	*Klebsiella spp*	Diabetes mellitus	23	12	19	3		NA
Lye et al. 1992	Urinary tract infection	SMX-TMP	*Klebsiella spp*	Diabetes mellitus	16	50	0	0		NA
Lye et al. 1992	Urinary tract infection	Cephalexin	*Escherichia coli*	Diabetes mellitus	16	16	0	0		NA
Lye et al. 1992	Urinary tract infection	Ampicilin	*Escherichia coli*	Diabetes mellitus	13	19	0	0		NA
Lye et al. 1992	Urinary tract infection	SMX-TMP	*Escherichia coli*	Diabetes mellitus	12	20	0	0		NA
Lye et al. 1992	Urinary tract infection	Ampicilin	*Klebsiella spp*	Diabetes mellitus	2	33	1	21		NA
Lye et al. 1992	Urinary tract infection	Ampicilin	*Klebsiella spp*	Diabetes mellitus	1	65	0	0		NA
Madaras et al. 2012	Community acquired Respiratory tract infection	non-pseudomonal third generation cephalosporins (ceftriaxone or cefotaxime) or non-pseudomonal 8-methoxy fluoroquinolones (moxifloxacin, gatifloxacin), the VA preferred agents for treatment of CAP	*Methicillin-resistant Staphylococcus aureus*	Diabetes mellitus	NI	NI	NI	NI	OR: 1.30 [0.60 ; 2.70]	NA

Madaras et al. 2012	Community acquired Respiratory tract infection	Non-pseudomonal third generation cephalosporins (ceftriaxone or cefotaxime) or non-pseudomonal 8-methoxy fluoroquinolones (moxifloxacin, gatifloxacin), the VA preferred agents for treatment of CAP	*Pseudomonas aeruginosa*	Diabetes mellitus	NI	NI	NI	NI	OR: 1.70 [1.00 ; 2.80]	Nursing home residence or discharge <=180 days prior to admission, positive MRSA status prior to admission, cephalosporin exposure <=365 days prior to admission, infusion therapy <=30 days prior to admission, direct ICU admission upon hospitalization
Madaras et al. 2012	Community acquired Respiratory tract infection	non-pseudomonal third generation cephalosporins (ceftriaxone or cefotaxime) or non-pseudomonal 8-methoxy fluoroquinolones (moxifloxacin, gatifloxacin), the VA preferred agents for treatment of CAP	*Methicillin-resistant Staphylococcus aureus and Pseudomonas aeruginosa*	Diabetes mellitus	46	70	72	187	OR: 2.20 [1.20 ; 4.30]	Nursing home residence or discharge <=180 days prior to admission, positive MRSA status prior to admission, Anti-pseudomonal fluoroquinolone exposure <=365 days prior to admission, 3rd generation cephalosporin exposure <=365 days prior to admission, Chronic inhaled corticosteroids
Malmartel et al. 2016	Urinary tract infection	Ofloxacin	*Escherichia coli, Enterococcus spp, Klebsiella spp, Proteus spp, Staphylococcus spp, Pseudomonas spp, Other bacteria*	Diabetes mellitus	29	95	41	205	OR: 0.50 [0.31 ; 0.82]	NA
Malmartel et al. 2016	Urinary tract infection	Co-trimoxazole	*Escherichia coli, Enterococcus spp, Klebsiella spp, Proteus spp, Staphylococcus spp, Pseudomonas spp, Other bacteria*	Diabetes mellitus	29	95	93	153	OR: 0.92 [0.37 ; 2.32]	NA
Malmartel et al. 2016	Urinary tract infection	Cefixim	*Escherichia coli, Enterococcus spp, Klebsiella spp, Proteus spp, Staphylococcus spp, Pseudomonas spp, Other bacteria*	Diabetes mellitus	11	113	12	234	OR: 1.11 [0.50 ; 2.49]	NA

Malmartel et al. 2016	Urinary tract infection	Nitrofurantoin	*Escherichia coli, Enterococcus spp, Klebsiella spp, Proteus spp, Staphylococcus spp, Pseudomonas spp, Other bacteria*	Diabetes mellitus	10	114	18	228	OR: 1.53 [0.89 ; 2.60]	NA
Malmartel et al. 2016	Urinary tract infection	Fosfomycin	*Escherichia coli, Enterococcus spp, Klebsiella spp, Proteus spp, Staphylococcus spp, Pseudomonas spp, Other bacteria*	Diabetes mellitus	7	117	15	231	OR: 1.90 [0.81 ; 4.43]	NA
Micek et al. 2015	Respiratory tract infection	Aminoglycosides, antipseudomonal carbapenems, antipseudomonal cephalosporins, antipseudomonal fluoroquinolones, antipseudomonal penicillins plus β-lactamase inhibitors, monobactams, phosphonic acids, and polymixins	*Pseudomonas aeruginosa*	Diabetes mellitus	79	137	130	338	OR: 1.90 [1.21 ; 3.00]	Age, sex, residence un communitity settings prior admission, residence in an inpatient rehabilitation facility prior to admission, antibiotics in the previous 30 days, COPD, solid tumor, dementia, ICU admission
Nakamura et al. 2013	All clinical isolates obtained from inpatients, except for faeces and nasal swabs	Piperacillin, ceftazidime, imipenem, amikacin, and levofloxacin	*MDR P. aeruginosa*	Diabetes mellitus	NI	NI	NI	NI	OR: 4.00 [2.40 ; 6.70]	Meropenem use, Urinary specimens, Fluoroquinolone use, ,Surgery, Length of hospital stay
Nuñez et al. 2020	Intra-abdominal infection	Ciprofloxacin (CIP) and ampicillin/sulbactam (AMS)	*Escherichia coli, Streptococcus spp., Klebsiella pneumoniae, Enterobacter spp., Pseudomonas spp., Enterococcus spp., Others (Gram-negative bacillia, Staphylococcus aureus, Candida spp.)*	Diabetes mellitus	NI	NI	NI	NI	OR: 3.60 [1.10 ; 11.60]	NI
Papazafiropoulou et al. 2009	Urinary tract infection	Ampicilin	*Escherichia coli*	Diabetes mellitus	50.064384	15.983616	323.1618	67.130224	PR: 0.67 [0.33 ; 1.35]	NA
Papazafiropoulou et al. 2009	Urinary tract infection	SMX-TMP	*Escherichia coli*	Diabetes mellitus	19.021824	47.026176	105.7691	284.522868	PR: 0.69 [0.34 ; 1.40]	NA

Carrillo-Larco RM, *et al. J Epidemiol Community Health* 2021; 76:75–84. doi: 10.1136/jech-2020-216029

Papazafiropoulou et al. 2009	Urinary tract infection	Ciprofloxacin	*Escherichia coli*	Diabetes mellitus	14.992896	51.055104	63.2273	327.064696	PR: 0.73 [0.39 ; 1.37]	NA
Papazafiropoulou et al. 2009	Urinary tract infection	Ciprofloxacin	*Pseudomonas*	Diabetes mellitus	7.348224	12.619776	46.81295	48.919052	PR: 0.75 [0.41 ; 1.39]	NA
Papazafiropoulou et al. 2009	Urinary tract infection	Imipenem	*Pseudomonas*	Diabetes mellitus	6.9888	12.9792	46	49.732	PR: 0.92 [0.79 ; 1.06]	NA
Papazafiropoulou et al. 2009	Urinary tract infection	Ceftazidime	*Pseudomonas*	Diabetes mellitus	5.9904	13.9776	41.73915	53.992848	PR: 1.06 [0.70 ; 1.61]	NA
Papazafiropoulou et al. 2009	Urinary tract infection	Amikacin	*Pseudomonas*	Diabetes mellitus	5.9904	13.9776	43.17513	52.556868	PR: 1.40 [0.85 ; 2.31]	NA
Patolia et al. 2018	Bacteriemia (central venous catheter, urinary catheter, Urinary tract infection, intra-abdominal, unknown)	MDR: (a) ESBL producing Enterobacteriaceae; (b) microorganisms with intrinsic resistance mechanisms such as Stenotrophomonas maltophilia, Burkholderia cepacia, and Ralstonia pickettii; and (c) any GNB (e.g. Acinetobacter spp., Enterobacteriaceae, and Pseudomonas spp.) resistant to three or more of the following drug class: piperacillin/tazobactam, cephalosporins (cefazolin, ceftriaxone, ceftazidime, and cefepime), carbapenems (imipenem), monobactams (aztreonam), aminoglycosides (gentamicin, tobramycin, and amikacin), and fluoroquinolones (ciprofloxacin and levofloxacin).	*Gram negative bacteria*	Diabetes mellitus	18	34	28	97	OR: 2.18 [1.00 ; 4.88]	Previous antibiotic use, Urinary catheter, Length of hospitalization before the onset of GNR bacteremia 72 h, Use of central venous catheter (⩾48 h) before the onset of GNR bacteremia
Pinheiro et al. 2010	Urinary tract infection	ESBL-Producing	*Enteric Gram-Negative Bacilli (Escherichia coli and Enterobacter sp.)*	Diabetes mellitus	4	10	11	20	PR: 0.81 [0.31 ; 2.09]	NA

Ramos-Lazaro et al. 2018	Urinary tract infection	Ceftriaxona	*Escherichia coli, Klebsiela spp.,, Enterobacter spp., Serratia spp., Citrobacter spp., Morganella spp., Proteus mirabilis, Pseudomona aeruginosa, , Staphylococcus spp., P. aeruginosa, Acinetobacter spp., Enterococcus spp., Candida spp.*	Diabetes mellitus	38	110	65	339	OR: 1.80 [1.14 ; 2.84]	NA
Randrianirina et al. 2010	Surgical wounds, depp pus, blood, urinary tract, respiratory trac and burn	Multiple resistant	*Escherichia coli, Klebsiella sp., or Staphylococcus spp., Proteus mirabilis, Pr. providencia - M. morganii, Enterobacter spp., Acinetobacter, Other GNB, Staphylococcus aureus, Coagulase neg. St., Enterococci, Streptococcus spp*	Diabetes mellitus	NI	NI	NI	NI	OR: 3.70 [1.30 ; 11.2]	NI: Variables giving p-values less than 0.20 in univariate analysis were analysed using multiple logistic regression.
Randrianirina et al. 2010	Surgical wounds, depp pus, blood, urinary tract, respiratory trac and burn infections	third-generation cephalosporins	*Enterobacteriaceae*	Diabetes mellitus	9	9	48	152	OR: 5.16 [1.35 ; 19.80]	NI: Variables giving p-values less than 0.20 in univariate analysis were analysed using multiple logistic regression.
Rogers et al. 2008	NI	NI	*methicillin-resistant Staphylococcus aureus (MRSA), aminoglycoside-resistant S aureus, vancomycin-resistant enterococci, and extended-spectrum beta-lactamase organisms,*	Diabetes mellitus	NI	NI	NI	NI	OR: 1.48 [1.26 ; 1.75]	State, age, sex, race, admitted from, devices at admission
Rogers et al. 2008	NI	NI	*Methicillin-resistant Staphylococcus aureus, aminoglycoside-resistant S aureus, vancomycin-resistant enterococci, and extended-spectrum beta-lactamase organisms,*	Diabetes mellitus	NI	NI	NI	NI	RR: 1.79 [1.54 ; 2.09]	NA

Romaniszyn et al. 2014	Any	Methicillin	*Staphylococcus aureus*	Diabetes mellitus	NI	NI	NI	NI	OR: 2.49 [0.98 ; 6.63]	NA
Sherchan et al. 2016	Urinary tract infection	ESBL-Producing	*Escherichia coli*	Diabetes mellitus	27	21	218	379	PR: 1.54 [1.17 ; 2.02]	NA
Terpenning et al. 1994	Urinary tract, skin and soft tissue infections	Gentamicin & ceftriaxone	*Methicillin-resistant Staphylococcus aureus*	Diabetes mellitus	NI	NI	NI	NI	OR: 5.10 [2.10 ; 18.60]	Underlying conditions (Recurrent Utls, Presence of wounds, Inflammatory bowel D., Prior pneumonia, Chronic renal D., Osteoarthritis), Katz functional scale, Devices, procedures (intermittent cath., gastrostomy tube), nutritional parameters (serum albumin)
Wu et al. 2014	Urinary tract infection	Levofloxacin	*Enterobacteriaceae pathogens (Escherichia coli , Klebsiella pneumoniae , Proteus spp. and Klebsiella oxytoca , E. Cloecae, K. oxytoca, M. morganil., S. marcescens., P. Vulgaris., Citrobacter)*	Diabetes mellitus	23	18	25	70	OR: 3.80 [1.50 ; 9.90]	Age, gender, recurrent UTI, prior hospitalization in the past 6 mo, prior antibiotic in the past 60d, urinary function abnormality, indwelling urinary catheter, old stroke, altered consciousness, urinary symptoms, chills, fever, hematuria
Zhang et al. 2019	Respiratory tract infection	Erythromycin	*Staphylococcus aureus*	Diabetes mellitus	97	33	136	57	PR: 0.86 [0.51 ; 1.43]	NA
Zhang et al. 2019	Respiratory tract infection	Methicillin	*Staphylococcus aureus*	Diabetes mellitus	94	50	124	97	PR: 0.96 [0.73 ; 1.25]	NA
Zhang et al. 2019	Respiratory tract infection	Levofloxacin	*Staphylococcus aureus*	Diabetes mellitus	83	47	104	89	PR: 0.97 [0.43 ; 2.18]	NA
Zhang et al. 2019	Respiratory tract infection	Clindamycin	*Staphylococcus aureus*	Diabetes mellitus	83	44	112	72	PR: 1.05 [0.8 ; 1.39]	NA
Zhang et al. 2019	Respiratory tract infection	Gentamicin	*Staphylococcus aureus*	Diabetes mellitus	70	61	83	111	PR: 1.06 [0.92 ; 1.21]	NA

Zhang et al. 2019	Respiratory tract infection	Ciprofloxacin	*Staphylococcus aureus*	Diabetes mellitus	46	28	43	51	PR: 1.07 [0.90 ; 1.27]	NA
Zhang et al. 2019	Respiratory tract infection	Tetraciclyne	*Staphylococcus aureus*	Diabetes mellitus	40	34	47	47	PR: 1.08 [0.81 ; 1.45]	NA
Zhang et al. 2019	Respiratory tract infection	Efazolin	*Staphylococcus aureus*	Diabetes mellitus	33	22	57	34	PR: 1.16 [0.98 ; 1.37]	NA
Zhang et al. 2019	Respiratory tract infection	Ampicillin/sulbactam	*Staphylococcus aureus*	Diabetes mellitus	32	21	55	41	PR: 1.18 [0.99 ; 1.42]	NA
Zhang et al. 2019	Respiratory tract infection	Moxifloxacin	*Staphylococcus aureus*	Diabetes mellitus	27	28	30	55	PR: 1.25 [0.99 ; 1.57]	NA
Zhang et al. 2019	Respiratory tract infection	Fosfomycin	*Staphylococcus aureus*	Diabetes mellitus	15	37	33	65	PR: 1.3 [0.19 ; 9.00]	NA
Zhang et al. 2019	Respiratory tract infection	Rifampicin	*Staphylococcus aureus*	Diabetes mellitus	9	119	14	180	PR: 1.35 [0.54 ; 3.42]	NA
Zhang et al. 2019	Respiratory tract infection	Sulfametoxazole	*Staphylococcus aureus*	Diabetes mellitus	8	122	9	189	PR: 1.36 [1.02 ; 1.80]	NA
Zhang et al. 2019	Respiratory tract infection	Nitrofurantoin	*Staphylococcus aureus*	Diabetes mellitus	2	72	0	89	PR: 1.39 [0.94 ; 2.06]	NA
Zhang et al. 2019	Respiratory tract infection	Quinupristin	*Staphylococcus aureus*	Diabetes mellitus	2	72	2	94		NA

NI: No information; NA: Not applicable

Supplementary Table 4: Case-control studies

Año y autor	Infection site	Antibiotic	Bacteria	Diabetes type	Diabetics with ant. resistance	Diabetics without ant. resistance	Non-diabetics with ant. resistance	Non-diabetics without ant. resistance	Measure of association	Adjustment
Anesi et al. 2016	Urinary tract infection	Extended-spectrum cephalosporin	*Extended-Spectrum Cephalosporin-Resistant Enterobacteriaceae: Escherichia coli (76%), Klebsiella species (13%), and Enterobacter species (9%).*	Diabetes mellitus	31	14	120	137	OR: 2.91 [1.32 ; 6.41]	Age, presentation to emergency department, trimethoprim sulfamethoxazole; receipt within prior 6months
Apisarnthanarak et al. 2007	Urinary tract (60), bloodstream (9), respiratory tract infection (4)	Quinolones, trimethoprim/sulfamethoxazole, or aminoglycosides	*Extended-spectrum b-lactamase-producing Escherichia coli*	Diabetes mellitus	19	18	27	28	OR: 1.09 [0.48 ; 2.52]	NA
Aswani et al. 2014	Urinary tract infection	Amikacin	*Escherichia coli*	Diabetes mellitus	35	146	26	98	OR: 0.90 [0.51 ; 1.60]	NA
Aswani et al. 2014	Urinary tract infection	Ampicilin	*Escherichia coli*	Diabetes mellitus	151	30	103	21	OR: 1.03 [0.56 ; 1.89]	NA
Aswani et al. 2014	Urinary tract infection	Augmentin	*Escherichia coli*	Diabetes mellitus	104	77	77	47	OR: 0.82 [0.52 ; 1.32]	NA
Aswani et al. 2014	Urinary tract infection	Aztreonam	*Escherichia coli*	Diabetes mellitus	141	40	96	28	OR: 1.03 [0.59 ; 1.78]	NA
Aswani et al. 2014	Urinary tract infection	Cefotaxime	*Escherichia coli*	Diabetes mellitus	101	80	79	45	OR: 0.72 [0.45 ; 1.15]	NA
Aswani et al. 2014	Urinary tract infection	Cefepime	*Escherichia coli*	Diabetes mellitus	92	89	79	45	OR: 0.59 [0.37 ; 0.94]	NA
Aswani et al. 2014	Urinary tract infection	Gentamycin	*Escherichia coli*	Diabetes mellitus	57	124	41	83	OR: 0.93 [0.57 ; 1.52]	NA
Aswani et al. 2014	Urinary tract infection	Cefoperazonesulbactum	*Escherichia coli*	Diabetes mellitus	27	154	24	100	OR: 0.73 [0.4 ; 1.34]	NA
Aswani et al. 2014	Urinary tract infection	Meropenem	*Escherichia coli*	Diabetes mellitus	11	170	6	118	OR: 1.27 [0.46 ; 3.54]	NA

Aswani et al. 2014	Urinary tract infection	Netilmycin	*Escherichia coli*	Diabetes mellitus	44	137	27	97	OR: 1.15 [0.67 ; 1.99]	NA
Aswani et al. 2014	Urinary tract infection	Norfloxacin	*Escherichia coli*	Diabetes mellitus	136	45	82	42	OR: 1.55 [0.94 ; 2.56]	NA
Aswani et al. 2014	Urinary tract infection	Piperacillin-Tazobactum	*Escherichia coli*	Diabetes mellitus	57	124	33	91	OR: 1.27 [0.76 ; 2.1]	NA
Aswani et al. 2014	Urinary tract infection	Cotrimoxazole	*Escherichia coli*	Diabetes mellitus	111	70	87	37	OR: 0.67 [0.41 ; 1.1]	NA
Aswani et al. 2014	Urinary tract infection	Ceftriaxone	*Escherichia coli*	Diabetes mellitus	91	90	68	56	OR: 0.83 [0.53 ; 1.32]	NA
Aswani et al. 2014	Urinary tract infection	Amikacin	*Klebsiella*	Diabetes mellitus	20	161	11	113	OR: 1.28 [0.59 ; 2.77]	NA
Aswani et al. 2014	Urinary tract infection	Ampicilin	*Klebsiella*	Diabetes mellitus	161	20	102	22	OR: 1.74 [0.9 ; 3.34]	NA
Aswani et al. 2014	Urinary tract infection	Augmentin	*Klebsiella*	Diabetes mellitus	80	101	65	59	OR: 0.72 [0.45 ; 1.14]	NA
Aswani et al. 2014	Urinary tract infection	Aztreonam	*Klebsiella*	Diabetes mellitus	112	69	79	45	OR: 0.92 [0.58 ; 1.48]	NA
Aswani et al. 2014	Urinary tract infection	Cefotaxime	*Klebsiella*	Diabetes mellitus	111	70	70	54	OR: 1.22 [0.77 ; 1.95]	NA
Aswani et al. 2014	Urinary tract infection	Cefepime	*Klebsiella*	Diabetes mellitus	83	98	63	61	OR: 0.82 [0.52 ; 1.3]	NA
Aswani et al. 2014	Urinary tract infection	Gentamycin	*Klebsiella*	Diabetes mellitus	50	131	43	81	OR: 0.72 [0.44 ; 1.18]	NA
Aswani et al. 2014	Urinary tract infection	Cefoperazonesulbactum	*Klebsiella*	Diabetes mellitus	23	158	7	117	OR: 2.43 [1.01 ; 5.86]	NA
Aswani et al. 2014	Urinary tract infection	Meropenem	*Klebsiella*	Diabetes mellitus	8	173	0	124	OR: 2.91 [1.32 ; 6.41]	NA
Aswani et al. 2014	Urinary tract infection	Netilmycin	*Klebsiella*	Diabetes mellitus	32	149	13	111	OR: 1.09 [0.48 ; 2.52]	NA
Aswani et al. 2014	Urinary tract infection	Norfloxacin	*Klebsiella*	Diabetes mellitus	121	60	81	43	OR: 0.90 [0.51 ; 1.60]	NA

Aswani et al. 2014	Urinary tract infection	Piperacillin-Tazobactum	*Klebsiella*	Diabetes mellitus	20	161	32	92	OR: 1.03 [0.56 ; 1.89]	NA
Aswani et al. 2014	Urinary tract infection	Cotrimoxazole	*Klebsiella*	Diabetes mellitus	111	70	86	38	OR: 0.82 [0.52 ; 1.32]	NA
Aswani et al. 2014	Urinary tract infection	Ceftriaxone	*Klebsiella*	Diabetes mellitus	94	87	70	54	OR: 1.03 [0.59 ; 1.78]	NA
Aswani et al. 2014	Urinary tract infection	Amikacin	*Pseudomonas*	Diabetes mellitus	11	170	31	93	OR: 0.72 [0.45 ; 1.15]	NA
Aswani et al. 2014	Urinary tract infection	Ampicilin	*Pseudomonas*	Diabetes mellitus	170	11	109	15	OR: 0.59 [0.37 ; 0.94]	NA
Aswani et al. 2014	Urinary tract infection	Augmentin	*Pseudomonas*	Diabetes mellitus	147	34	93	31	OR: 0.93 [0.57 ; 1.52]	NA
Aswani et al. 2014	Urinary tract infection	Aztreonam	*Pseudomonas*	Diabetes mellitus	136	45	78	46	OR: 0.73 [0.4 ; 1.34]	NA
Aswani et al. 2014	Urinary tract infection	Cefotaxime	*Pseudomonas*	Diabetes mellitus	136	45	109	15	OR: 1.27 [0.46 ; 3.54]	NA
Aswani et al. 2014	Urinary tract infection	Cefepime	*Pseudomonas*	Diabetes mellitus	119	62	71	53	OR: 1.15 [0.67 ; 1.99]	NA
Aswani et al. 2014	Urinary tract infection	Gentamycin	*Pseudomonas*	Diabetes mellitus	79	102	40	84	OR: 1.55 [0.94 ; 2.56]	NA
Aswani et al. 2014	Urinary tract infection	Cefoperazonesulbactum	*Pseudomonas*	Diabetes mellitus	56	125	47	77	OR: 1.27 [0.76 ; 2.10]	NA
Aswani et al. 2014	Urinary tract infection	Meropenem	*Pseudomonas*	Diabetes mellitus	23	158	16	108	OR: 0.67 [0.41 ; 1.10]	NA
Aswani et al. 2014	Urinary tract infection	Netilmycin	*Pseudomonas*	Diabetes mellitus	38	143	0	124	OR: 0.83 [0.53 ; 1.32]	NA
Aswani et al. 2014	Urinary tract infection	Norfloxacin	*Pseudomonas*	Diabetes mellitus	152	29	78	46	OR: 1.28 [0.59 ; 2.77]	NA
Aswani et al. 2014	Urinary tract infection	Piperacillin-Tazobactum	*Pseudomonas*	Diabetes mellitus	34	147	47	77	OR: 1.74 [0.90 ; 3.34]	NA
Aswani et al. 2014	Urinary tract infection	Cotrimoxazole	*Pseudomonas*	Diabetes mellitus	147	34	109	15	OR: 0.72 [0.45 ; 1.14]	NA

Aswani et al. 2014	Urinary tract infection	Ceftriaxone	*Pseudomonas*	Diabetes mellitus	140	41	89	35	OR: 0.92 [0.58 ; 1.48]	NA
Borer et al. 2012	Any	Carbapenems	*Klebsiella pneumoniae*	Diabetes mellitus	19.95	122.032	22.05	341.968	OR: 1.22 [0.77 ; 1.95]	Not clear
Briongos-Figuero et al. 2012	Urinary tract infection	Amoxycillin / clavulanic acid, Carbapenem, Gentamicin, Amikacin, Trimethoprim-sulphamethoxazole, Fosfomycin, Ciprofloxacin, Nitrofurantoin, Nalidixic acid	*Escherichia coli*	Diabetes mellitus	35	23	62	80	OR: 0.82 [0.52 ; 1.30]	NA
Chitnis et al. 2012	Any	Carbapenems	*Enterobacteriae*	Diabetes mellitus	15	7	19	27	OR: 0.72 [0.44 ; 1.18]	Sex, lenght of stay prior to index date, ventilator days, hemodialisis, TPN-days, invasive procedures, Charlstone score, contact precautions,
Colodner et al. 2008	Urinary tract infection	Ciprofloxacin and ofloxacin	*Escherichia coli*	Type 2 Diabetes mellitus	40	15	110	135	OR: 2.43 [1.01 ; 5.86]	NA
Colodner et al. 2004	Urinary tract infection	Extended-spectrum beta-lactamases	*Escherichia coli or Klebsiella*	Diabetes mellitus	43	18	85	165	OR: 2.57 [1.20 ; 5.51]	Age, E.coli infection, Klebsiella infection, duration of atb treatment, hospitalization in the last 3 months, gender, age more than 60, underlying diseases (cardiovascular, gastrointestinal, genitourinary, recurrent UTI, neurological, malignancies)
Dan et al. 2016	Bloodstream (Urinary tract infection , others)	Fluoroquinolone	*Gram negative*	Diabetes mellitus	61	236	82	445	OR: 1.54 [1.03 ; 2.28]	Age, sex, Indwelling urinary catheter, recent outpatient procedure, Residence at skilled nursing facility, recent hospitalization, prior flouroquinolone use

Dodds Ashley et al. 2004	Postoperative Mediastinitis	Methiciline	*Staphylococcus aureus*	Diabetes mellitus	37	22	27	57	OR: 3.55 [1.77 ; 7.14]	NA
García-Tello et al. 2017	Urinary tract infection	Extended-spectrum beta-lactamases	*Escherichia coli, Klebsiella sp., Enterobacteriaceae*	Diabetes mellitus	150	238	266	870	OR: 2.06 [1.61 ; 2.64]	NA
Hayakawa et al. 2013	Urinary tract, bloodstream, wound infections	Vancomycin	*Enterococcus faecalis*	Diabetes mellitus	281	209	251	323	OR: 1.73 [1.36 ; 2.21]	NA
Hershow et al. 1998	Bloodstream	ciprofloxacin	*Methicillin resistant staphilococcus aureus*	Diabetes mellitus	9	0	6	10		NA
Hershow et al. 1998	Respiratory & bone infections	ciprofloxacin	*Methicillin resistant staphilococcus aureus*	Diabetes mellitus	19	4	37	10	OR: 1.28 [0.36 ; 4.64]	NA
Hsu et al. 2005	Respiratory, urinary, wound, bloodstream infections	fluoroquinolones	*Pseudomonas aeruginosa*	Diabetes mellitus	31	9	60	77	OR: 6.4 [2.10 ; 19.30]	Residence site, Prior FQ exposure
Isendhal et al. 2019	Bloodstream	Extended-spectrum b-lactamase-producing Enterobacteriaceae (Aminopenicillins including b-lactamase inhibitor combinations (but excluding pivmecillinam), trimethoprim, trimethoprim-sulfamethoxazole, fluoroquinolones and cephalosporins)	*Enterobacteriaceae*	Diabetes mellitus	209	514	736	8876	OR: 2.03 [1.58 ; 2.61]	Charlson Index score, urological disorders and hospitalization 8e91 days before index date.
Jinnah et al. 1996	Urinary tract infection	Amoxicilin	*Escherichia coli*	Diabetes mellitus	16	9	22	3	OR: 0.24 [0.06 ; 1.04]	NA
Jinnah et al. 1996	Urinary tract infection	Amoxicilin	*Escherichia coli*	Diabetes mellitus	15	0	22	3		NA
Jinnah et al. 1996	Urinary tract infection	Cloxacilin	*Escherichia coli*	Diabetes mellitus	11	14	16	9	OR: 0.44 [0.14 ; 1.38]	NA
Jinnah et al. 1996	Urinary tract infection	Cloxacilin	*Escherichia coli*	Diabetes mellitus	15	0	16	9		NA
Jinnah et al. 1996	Urinary tract infection	Erythromysin	*Escherichia coli*	Diabetes mellitus	14	11	16	9	OR: 0.72 [0.23 ; 2.23]	NA
Jinnah et al. 1996	Urinary tract infection	Erythromysin	*Escherichia coli*	Diabetes mellitus	14	1	16	9	OR: 7.88 [0.88 ; 70.15]	NA

Jinnah et al. 1996	Urinary tract infection	Cephalexin	*Escherichia coli*	Diabetes mellitus	13	12	13	12	OR: 1.00 [0.33 ; 3.03]	NA
Jinnah et al. 1996	Urinary tract infection	Cephalexin	*Escherichia coli*	Diabetes mellitus	14	1	13	12	OR: 12.92 [1.47 ; 113.78]	NA
Jinnah et al. 1996	Urinary tract infection	Gentamycin	*Escherichia coli*	Diabetes mellitus	6	19	5	20	OR: 1.26 [0.33 ; 4.84]	NA
Jinnah et al. 1996	Urinary tract infection	Gentamycin	*Escherichia coli*	Diabetes mellitus	7	8	5	20	OR: 3.5 [0.85 ; 14.34]	NA
Jinnah et al. 1996	Urinary tract infection	Ciprofloxacin	*Escherichia coli*	Diabetes mellitus	1	24	1	24	OR: 1.00 [0.06 ; 16.93]	NA
Jinnah et al. 1996	Urinary tract infection	Ciprofloxacin	*Escherichia coli*	Diabetes mellitus	6	9	1	24	OR: 16.00 [1.68 ; 152.02]	NA
Khurram et al. 2007	Any	Methiciline	*Staphylococcus aureus*	Diabetes mellitus	16	2	21	18	OR: 6.857	NA
Kim et al. 2014	Respiratory tract infection	Carbapenems (meropenem, imipenem)	*Gram-negative bacteria or Stenotrophomonas maltophilia*	Diabetes mellitus	22	17	60	105	OR: 2.82 [1.25 ; 6.38]	Neurological disease; Macabe and Jackson classification; ventilator associated pneumonia; APACHE II; Radiologic score; prior antibiotic usage
Manzur et al. 2007	Bloodstream (Unknown, soft tissue, catheter, osteo-articular, Urinary tract infection , endocarditis, endovascular, others)	Methiciline	*Staphylococcus aureus*	Diabetes mellitus	21	32	29	66	OR: 1.49 [0.74 ; 3.00]	NA
Park et al. 2015	Urinary tract infection	Amikacin, Gentamicin, Tobramycin, Ciprofloxacin, Levofloxacin, Amoxicillun-clavulanate, Piperacillin-tazobactam, trimethoprim-sulfamethoxazole, fluoroquinolones, aminoglycosides.	*Escherichia coli*	Diabetes mellitus	27	53	48	172	OR: 1.69 [0.78 ; 3.44]	age >55, sex, urinary tract abnormalities, aqcute pielonefritis recurrence and antibiotic use within the previous year

Ren et al. 2019	Respiratory tract infection	Pan resistant (cefotaxime, piperacillin, imipenem, gentamicin, levofloxacin, tetracycline, ceftazidime, polymyxin)	*Acinetobacter baumannii*	Diabetes mellitus	24	4	74	45	OR: 5.98 [2.87 ; 14.60]	Not clear
Saade et al. 2016	Genitourinary and bloodstream	fluoroquinolones	*Escherichia coli*	Diabetes mellitus	168	16940	260	42101	OR: 1.50 [1.20 ; 1.80]	Age, history of culture positive for FQ-resistant Escherichia coli, admission in te past, FQ use in the past year, other antibiotic (penicillins, cephalosporins, macrolides, trimethoprim-sulfamethoxazole, tetracyclines, and aminoglycosides) use in the past year
Saibal et al. 2012	Respiratory tract infection	Co-amoxiclav	*Klepsiella pneumoniae*	Diabetes mellitus	9	0	0	2		NA
Saibal et al. 2012	Respiratory tract infection	Ceftriaxone	*Klepsiella pneumoniae*	Diabetes mellitus	1	8	0	2		NA
Saibal et al. 2012	Respiratory tract infection	Ceftazidime	*Klepsiella pneumoniae*	Diabetes mellitus	1	8	0	2		NA
Saibal et al. 2012	Respiratory tract infection	Clarithromycin	*Klepsiella pneumoniae*	Diabetes mellitus	8	1	0	2		NA
Saibal et al. 2012	Respiratory tract infection	Levofloxcin	*Klepsiella pneumoniae*	Diabetes mellitus	7	2	1	1	OR: 3.5 [0.14 ; 84.7]	NA
Saibal et al. 2012	Respiratory tract infection	Meropenem	*Klepsiella pneumoniae*	Diabetes mellitus	0	9	2	0		NA
Saibal et al. 2012	Respiratory tract infection	Imipenem	*Klepsiella pneumoniae*	Diabetes mellitus	0	9	2	0		NA
Saibal et al. 2012	Respiratory tract infection	Co-amoxiclav	*Staphylococcus aureus*	Diabetes mellitus	2	0	1	0		NA
Saibal et al. 2012	Respiratory tract infection	Ceftriaxone	*Staphylococcus aureus*	Diabetes mellitus	0	2	0	1		NA

Saibal et al. 2012	Respiratory tract infection	Ceftazidime	*Staphylococcus aureus*	Diabetes mellitus	1	1	0	1		NA
Saibal et al. 2012	Respiratory tract infection	Clarithromycin	*Staphylococcus aureus*	Diabetes mellitus	1	1	0	1		NA
Saibal et al. 2012	Respiratory tract infection	Levofloxcin	*Staphylococcus aureus*	Diabetes mellitus	1	1	1	0		NA
Saibal et al. 2012	Respiratory tract infection	Meropenem	*Staphylococcus aureus*	Diabetes mellitus	0	2	1	0		NA
Saibal et al. 2012	Respiratory tract infection	Imipenem	*Staphylococcus aureus*	Diabetes mellitus	0	2	1	0		NA
Silva et al. 2006	Any	Extended-spectrum beta-lactamases	*Klebsiella pneumoniae*	Diabetes mellitus					OR: 5.09	Not clear
Soraas et al. 2013	Urinary tract infection	Extended-spectrum beta-lactamases (Mecillinam, Macrolides, Tetracyclines, Fluoroquinolones, Nitrofurantoin, Trimethoprim or trimethoprim/sulfamethoxazole, b-lactams except mecillinam, Methenamine hippurate)	*Escherichia coli or Klebsiella*	Diabetes mellitus	12	9	88	181	OR: 3.20 [1.00 ; 11.00]	Not clear
Vinken et al. 2018	Urinary tract infection	Nitrofurantoin, trimethoprim, fosfomycin, ciprofloxacin, amoxicillin/clavulanic acid and/or trimethoprim/sulfamethoxazole	*Enterococcus spp*	Type 2 Diabetes mellitus	32	0	31	0		NA
Vinken et al. 2018	Urinary tract infection	Nitrofurantoin, trimethoprim, fosfomycin, ciprofloxacin, amoxicillin/clavulanic acid and/or trimethoprim/sulfamethoxazole	*Escherichia coli*	Type 2 Diabetes mellitus	84	130	77	124	OR: 1.04 [0.7 ; 1.54]	NA

Vinken et al. 2018	Urinary tract infection	Nitrofurantoin, trimethoprim, fosfomycin, ciprofloxacin, amoxicillin/clavulanic acid and/or trimethoprim/sulfamethoxazole	*Klebsiella oxytoca*	Type 2 Diabetes mellitus	7	3	9	2	OR: 0.52 [0.07 ; 4.00]	NA	
Vinken et al. 2018	Urinary tract infection	Nitrofurantoin, trimethoprim, fosfomycin, ciprofloxacin, amoxicillin/clavulanic acid and/or trimethoprim/sulfamethoxazole	*Klebsiella pneumoniae*	Type 2 Diabetes mellitus	28	2	25	3	OR: 1.68 [0.26 ; 10.89]	NA	
Vinken et al. 2018	Urinary tract infection	Nitrofurantoin, trimethoprim, fosfomycin, ciprofloxacin, amoxicillin/clavulanic acid and/or trimethoprim/sulfamethoxazole	*Other (22 species)*	Type 2 Diabetes mellitus	24	1	30	4	OR: 3.20 [0.34 ; 30.55]	NA	
Vinken et al. 2018	Urinary tract infection	Nitrofurantoin, trimethoprim, fosfomycin, ciprofloxacin, amoxicillin/clavulanic acid and/or trimethoprim/sulfamethoxazole	*Proteus mirabilis*	Type 2 Diabetes mellitus	20	0	24	0		NA	
Vinken et al. 2018	Urinary tract infection	Nitrofurantoin, trimethoprim, fosfomycin, ciprofloxacin, amoxicillin/clavulanic acid and/or trimethoprim/sulfamethoxazole	*Pseudomonas aeruginosa*	Type 2 Diabetes mellitus	8	0	12	0		NA	
Vinken et al. 2018	Urinary tract infection	Nitrofurantoin, trimethoprim, fosfomycin, ciprofloxacin, amoxicillin/clavulanic acid and/or trimethoprim/sulfamethoxazole	*Escherichia coli, Enterococcus spp, Klebsiella pneumoniae, Proteus mirabilis, Klebsiella oxytoca, Pseudomonas aeruginosa, an others 32 species*	Type 2 Diabetes mellitus	114	169	124	159	OR: 0.86 [0.62 ; 1.21]	NA	

Vinken et al. 2018	Urinary tract infection	Nitrofurantoin, trimethoprim, fosfomycin, ciprofloxacin, amoxicillin/clavulanic acid and/or trimethoprim/sulfamethoxazole	*Escherichia coli, Enterococcus spp, Klebsiella pneumoniae, Proteus mirabilis, Klebsiella oxytoca, Pseudomonas aeruginosa, an others 32 species*	Type 2 Diabetes mellitus		OR: 0.90 [0.40 ; 1.80]	Age ≥75 years, hosptal admmission in preceding 12mo, Elderly home resident, Use of antibiotics in preceding 12 mo, UTI in preceding 12mo, Micturition complaints in preceding 6w

Wright et al. 2000	Urinary tract infection	Any (Pseudomonas, Enterococcus, and Staphylococcus species undergo susceptibility testing using different antimicrobials because of inherently distinct susceptibility patterns. Pseudomonas species are tested using aminoglycosides, fluoroquinolones, and beta-laetam antipseudomonal antibiotics. Because Pseudomonas species are inherently resistant to ampicillin, amoxicillin/clavulanate, nitrofurantoin, and TMPSMX, all Pseudomonas infections were considered multiply resistant. Enterococcus species were tested for susceptibility to ampicillin, nitrofurantoin, ofloxacin, and vancomycin. Enterococcus species are intrinsically resistant to TMPSMX. Therefore, Enterococcus isolates resistant to at least one other of the tested antibiotics were considered multiply resistant. Staphylococcus species were considered multiply resistant if resistant to at least two of the following: ampicillin, TMP-SMX, amoxicillin/clavulanate, ofloxacin, or methicillin.)	*Any (Escherichia coil, Proteus mirabilis, Klebsiella pneumoniae, Pseudomonas aeruginosa, Enterobactersp, Citrobactersp., Staphylococcus sp. [S saprophyticus, S epidermidis, S aureus], Enterococcus sp., Streptococcus sp.)*	Diabetes mellitus	16	25	102	292	OR: 1.83 [0.94 ; 3.57]	NA

Wright et al. 1999	Urinary tract infection	TMP-SMX	*Coliform (Escherichia coli, Proteus mirabilis, Klebsiella pneumoniae, Enterobacter species, Citrobacter freundi, Providencia species, and Morganella morganii)*	Diabetes mellitus	9	19	58	362	OR: 3.10 [1.20 ; 8.40]	Age, use of catheter, history of recurrent UTI, urologic abnormality, neurologic abnormality, recently in hospital, current atb, current TMP-SMX

NI: No information; NA: Not applicable

Supplementary Table 5: Risk of bias

Study Design	Author	Newcastle-Ottawa Quality Assessment Scale							
		Selection 1	Selection 2	Selection 3	Selection 4	Comparability	Exposure 1	Exposure 2	Exposure 3
Case-Control	Anesi et al. 2016	★	★	★	★	★★	★	★	
Case-Control	Apisarnthanarak et al. 2007	★	★	★	★	★	★	★	
Case-Control	Aswani et al. 2014	★	★	★	★	★	★	★	
Cross-sectional	Bailey et al. 2013	★	★	★	NA		★	NA	NA
Cohort	Baillargeon et al. 2004		★	★	★		★	★	★
Cross-sectional	Benenson et al. 2005	★	★	★	NA		★	NA	NA
Cross-sectional	Bonadio et al. 2006	★	★	★	NA		★	NA	NA
Case-Control	Borer et al. 2012	★	★	★	★	★★		★	★
Case-Control	Briongos-Figuero et al. 2012	★	★	★	★	★	★	★	
Cross-sectional	Chen et al. 2013	★	★	★	NA	★	★	NA	NA
Cross-sectional	Chiquet et al. 2015	★	★	★	NA		★	NA	NA
Case-Control	Chitnis et al. 2012	★	★	★	★	★★	★	★	★
Cross-sectional	Chiu et al. 2017	★	★	★	NA	★	★	NA	NA
Cross-sectional	Chong et al. 2013		★	★	NA		★	NA	NA
Case-Control	Colodner et al. 2004	★	★	★	★	★★		★	★
Case-Control	Colodner et al. 2008	★	★	★	★		★	★	★
Case-Control	Dan et al. 2016	★	★	★	★	★★	★	★	★
Case-Control	Dodds Ashley et al. 2004	★	★	★	★		★	★	★
Case-Control	García-Tello et al. 2017	★	★	★	★		★	★	★
Case-Control	Hayakawa et al. 2013	★	★	★	★	★★	★	★	
Case-Control	Hershow et al. 1998	★	★	★	★		★	★	
Cross-sectional	Ho et al. 2019	★	★	★	NA	★	★	NA	NA
Case-Control	Hsu et al. 2005	★	★	★	★	★	★	★	★
Case-Control	Isendhal et al. 2019	★	★	★	★	★★	★	★	★
Cross-sectional	Jaaskelainen et al. 2017	★	★	★	NA		★	NA	NA
Case-Control	Jinnah et al. 1996	★	★	★	★		★	★	★
Case-Control	Khurram et al. 2007	★	★	★	★		★	★	★
Case-Control	Kim et al. 2014	★	★	★	★	★★	★	★	★
Cross-sectional	Kistler et al. 2019	★	★	★	NA		★	NA	NA
Cross-sectional	Kurup et al. 2019	★	★	★	NA		★	NA	NA
Cohort	Laupland et al. 2008	★	★	★	★		★	★	★

Cross-sectional	Levin et al. 2010		★	★	NA		★	NA	NA
Cross-sectional	Libert et al. 2008	★	★	★	NA		★	NA	NA
Cross-sectional	Liu et al. 2019	★	★	★	NA	★	★	NA	NA
Cross-sectional	Lye et al. 1992	★	★		NA		★	NA	NA
Cross-sectional	Madaras et al. 2012	★	★		NA	★	★	NA	NA
Cross-sectional	Malmartel 2016	★	★	★	NA	★	★	NA	NA
Case-Control	Manzur et al. 2007	★	★	★	★		★	★	★
Cross-sectional	Micek et al. 2015	★	★	★	NA	★★	★	NA	NA
Cross-sectional	Nakamura et al. 2013	★	★	★	NA	★	★	NA	NA
Cross-sectional	Nuñez_2016	★	★	★	NA		★	NA	NA
Cross-sectional	Papazafiropoulou et al. 2009		★	★	NA		★	NA	NA
Case-Control	Park et al. 2015	★	★	★	★	★★	★	★	
Cross-sectional	Patolia et al. 2018	★	★	★	NA	★★	★	NA	NA
Cohort	Pinheiro et al. 2010		★				★	★	★
Cross-sectional	Ramos-Lazaro et al. 2018	★	★	★	NA		★	NA	NA
Cross-sectional	Randrianirina et al. 2010	★	★	★	NA	★	★	NA	NA
Case-Control	Ren et al. 2019	★	★	★	★			★	★
Cohort	Rogers et al. 2008	★	★	★	★	★★	★	★	★
Cohort	Romaniszyn et al. 2014	★	★	★	★		★	★	★
Case-Control	Saade et al. 2016	★	★	★		★★	★	★	
Case-Control	Saibal et al. 2012	★	★	★	★		★	★	★
Cross-sectional	Sherchan et al. 2016	★	★		NA		★	NA	NA
Case-Control	Silva et al. 2006	★	★	★	★				
Case-Control	Soraas et al. 2013	★	★	★	★			★	★
Cross-sectional	Terpenning et al. 1994	★	★	★	NA	★★	★	NA	NA
Case-Control	Vinken et al. 2018		★	★	★	★	★	★	★
Case-Control	Wright et al. 1999	★	★	★	★	★★		★	★
Case-Control	Wright et al. 2000	★	★	★	★			★	★
Cross-sectional	Wu et al. 2014	★	★		NA	★★	★	NA	NA
Cross-sectional	Zhang et al. 2019	★	★	★	NA		★	NA	NA

NA: Not applicable

Supplementary Table 6: Characteristics of cross-sectional and cohort reports (expanded from Table 1 in the main paper)

Author	Country	Year of data collection	Study design	Sample based	Participant selection	Population description	Sample size	Age	% men
Bailey et al. 2013	USA	2010	Cross-sectional	Hospital-based	Consecutive patients	Patients aged ≥18 years who were evaluated and discharged from the emergency department with a discharge diagnosis of a community acquired urinary tract infection and a positive urine culture for *Escherichia coli.*	222	NI	18.47
Baillargeon et al. 2004	USA	2000	Cohort	Captive population-based	NI	Inmates incarcerated in the TDCJ prison system for any duration from 1 January 1999 through 31 December 2001.	299179	NI	88.84
Benenson et al. 2005	Egypt	2001	Cross-sectional	Hospital-based	Consecutive patients	All patients who were admitted through the emergency department and were found to have a positive blood culture drawn in the ED on admission.	131	69.00 ± 25.00	49.00
Bonadio et al. 2006	Italy	1999	Cross-sectional	Hospital-based	Consecutive patients	Patients who were over the age of 50 who were admitted to the department of medicine of the Pisa University-Hospital were screened for asymptomatic bacteriuria.	1321	72.96 ± 22.00	31.00
Chen et al. 2013	Taiwan	2010	Cross-sectional	Hospital-based	Consecutive patients	Hospitalized adults identified by a discharge diagnosis of urinary tract infection.	420	63.5 ± 13.97	24.60
Chiquet et al. 2015	France	2008	Cross-sectional	Hospital-based	NI	Post-cataract surgery endophthalmitis cases infected with Coagulase-negative staphylococci species.	68	76.20 ± 11.40	44.12
Chiu et al. 2017	Taiwan	2015	Cross-sectional	Hospital-based	NI	Patients age ≥18 years admitted through the emergency department for a urinary tract infection diagnosis; with symptoms of acute cystitis (frequency, dysuria, urgency, haematuria, or suprapubic pain), signs of acute pyelonephritis, body temperature 38.3°C with or without chills, flank pain, and costophrenic angle knocking tenderness, or symptomatic patients with an indwelling urinary catheter; and pyuria.	457	71.9	32.95
Chong et al. 2013	Singapore	2000	Cross-sectional	Hospital-based	Consecutive patients	Patients who had hand infections and underwent surgical debridement or amputation.	100	59.36	NI
Chong et al. 2013	Singapore	2009	Cross-sectional	Hospital-based	Consecutive patients	Patients who had hand infections and underwent surgical debridement or amputation	98	49.06	NI
Ho et al. 2019	Singapore	2016	Cross-sectional	Hospital-based	NI	Adult male and female clinic patients aged 21 years and above and identified by the physician to have urinary tract	299	60.80 ± 17.30	13.38

						infection-related symptoms were invited to participate and referred to a research assistant stationed on site.			
Jaaskelainen et al. 2017	Finland and Sweden	2010	Cross-sectional	Hospital-based	Consecutive patients	Patients with an infection affecting deeper soft tissue, infection that required significant surgical intervention, developed on a lower extremity in a subject with diabetes mellitus or peripheral vascular disease, or to have a major abscess or infected ulcer. In addition, the patient also had to have at least 1 systemic sign of infection (temperature >38 or <36°C, white blood cell count >10 000/mm3 or <4000/mm3). Patients with diabetic foot infection were excluded.	390	66.14 ± 18.60	58.21
Kistler et al. 2019	USA	2010	Cross-sectional	Hospital-based	Consecutive patients	Patients aged 18 to 89 with a history of a culture-positive hand nosocomial infection.	815	49.54	NI
Kurup et al. 2019	Guyana	2017	Cross-sectional	Hospital-based	Convenient and purposive	New patients with known foot ulcer, presence of bacteria, older than 18 years, willing to participate and were available during the sample/data collection period.	183	55.84 ± 15.68	50.82
Laupland et al. 2008	Canada	2003	Cohort	Hospital-based	Consecutive patients	All persons who resided in the Calgary Health Region and who developed bacteremic *S. aureus* infection between 1 January 2000 and 31 December 2006.	1542	61.70 ± 22.19	62.00
Levin et al. 2010	Canada and Israel	2003 and 2005	Cross-sectional	Hospital-based	Consecutive patients	Patients admitted to the intense care unit for > 48 h.	423	56.00 ± 21.97	60.28
Libert et al. 2008	Belgium	2003	Cross-sectional	Hospital-based	Consecutive patients	All patients admitted to Brugmann University Hospital, who presented an episode of *S. aureus* bloodstream infection.	154	63.2 ± 56.9	52.60
Liu et al. 2019	China	2017	Cross-sectional	Hospital-based	Consecutive patients	Inpatients of *Klebsiella pneumoniae* pneumonia with and without diabetes in a Hospital from China.	456	64.00 ± 15.72	72.15
Lye et al. 1992	Singapore	1989	Cross-sectional	Hospital-based	Consecutive patients	All case records of patients with nosocomial urinary tract infections at the National University hospital, Singapore with positive urine cultures were reviewed.	348	NI	NI

Madaras et al. 2012	USA	2006	Cross-sectional	Hospital-based	Consecutive patients	Patients with healthcare associated pneumonia with urinary tract infection. Subjects were identified by the following pneumonia-related discharge International Classification of Diseases (ICD-9 CM) codes: 1) a primary diagnosis of 480-483; 485-487.0 (pneumonia); or 2) a primary diagnosis of 507.0 (pneumonitis), 518.8 (respiratory failure), or 0.38 (septicaemia), and a secondary diagnosis of 480-483; 485-487.0.4 Eligibility required that patients received antibiotic therapy for pneumonia within 24 hours of admission, continue inpatient treatment for >24 hours, and meet any of the following guideline-defined criteria: 1) hospitalization during the preceding 90 days; 2) admission from a nursing home; 3) outpatient or home wound care, outpatient or home infusion therapy, or chronic haemodialysis. In addition, patients not meeting guideline-defined criteria, who had frequent healthcare system exposure, defined as 12 Emergency Department, Medicine, or Surgery clinic visits within 90 days of admission, were also included. Patients were excluded if they were directly transferred from another hospital, or had pneumonia-related ICD-9 codes but received inpatient care for pneumonia in a non-Veterans-Affairs hospital.	375	71.20 ± 12.40	98.67
Malmartel 2016	France	2014	Cross-sectional	Hospital-based	Consecutive patients	Patients aged over 18 years from ambulatory medical laboratories in urban and semi-rural places of the Parisian area.	1119	59.14 ± 19.96	26.22
Micek et al. 2015	USA<br>France<br>Germany<br>Italy<br>Spain	2002	Cross-sectional	Hospital-based	Consecutive patients	Patients with nosocomial pneumonia aged ≥18 years admitted for their index hospitalization within 36 months prior to study initiation. All eligible patients met a clinical diagnosis of nosocomial pneumonia defined as new or progressive infiltrates consistent with pneumonia on chest radiograph or computed tomography and either a temperature >38.3°C or leucocytosis >10,000 cells/mm3 or both.	740	59.47 ± 16.61	67.97
Nakamura et al. 2013	Japan	2003	Cross-sectional	Hospital-based	Consecutive patients	Microbiological data of all clinical isolates obtained from inpatients, except for faeces and nasal swabs.	740	NI	NI

Nuñez et al. 2020	Argentina	2016	Cross-sectional	Hospital-based	NI	Adult patients who underwent intra-abdominal surgery involving the peritoneal cavity with at least one positive aerobic isolate from intra-abdominal samples (peritoneum or visceral samples, including both hollow and solid viscera).	119	54 ± 21	41.18
Papazafiropoulou et al. 2009	Greece	2008	Cross-sectional	Hospital-based	Consecutive patients	Urine culture specimens from patients hospitalized for urinary tract infections between 1st of January 2007 up to 30th November 2008.	1244	72.30 ± 12.60	31.35
Patolia et al. 2018	USA	2014	Cross-sectional	Hospital-based	Consecutive patients	Patients aged 18 years and older with at least one episode of Gram Negative bacteria bacteraemia during a hospital admission were included in the study. There were no other exclusion criteria.	177	±	57.00
Pinheiro et al. 2010	Brazil	2004 and 2007	Cohort	Hospital-based	Consecutive patients	Kidney recipients transplanted between January 2003 and December 2005, and those between January 2006 and December 2008 with urinary tract infection. The presence of a urinary tract infection episode was based on clinical, microbiological data and response to antimicrobial treatment.	45	42.00 ± 14.50	68.89
Ramos-Lazaro et al. 2018	Spain	2011	Cross-sectional	Hospital-based	NI	Males 18 years old or older with community-acquired urinary tract infection in urgency room.	552	66.00 ± 17.00	100.00
Randrianirina et al. 2010	Madagascar	2007	Cross-sectional	Hospital-based	Consecutive patients	Patients with nosocomial infection which was defined as any infection that occurred more than 48 hours after admission of the patient to hospital. Any patient who had an infection on admission and had not recovered from that episode the day of the survey and any inpatient who had been hospitalized for fewer than three days was not considered a nosocomial infection case.	651	37.60 ± 19.64	59.40
Rogers et al. 2008	USA	2003	Cross sectional and Cohort	Captive population-based	NI	Residents who were newly admitted to skilled nursing facilities in California, Florida, Michigan, New York, and Texas. Only long-term residents (ie, those remaining in the facility for an entire year) were included.	56182	NI	30.79

Romaniszyn et al. 2014	Poland	2010	Cohort	Captive population-based	Consecutive patients	Residents of long-term care facilities (Nursing home and residential homes). Nursing home infection was defined as an institutionary tract infection on where residents need 24 h/day medical or skilled nurses' supervision and provide more intensive health care than residential homes, where residents are unable to live independently and require supervision or assistance with the activities of daily living. A resident was defined as a person who has stayed in long-term care facilities for longer than 48 hours at the time of the study. Residents with mental disorders and residents younger than 65 years were not included in the study.	193	79.90 ± 11.60	39.90
Sherchan et al. 2016	Nepal	2015	Cross-sectional	Hospital-based	Consecutive patients	Urine sample from outpatients and inpatients collected between June 2014 to May 2015 from which *Escherichia coli* was isolated (with special reference to extended-spectrum β-lactamase) was included in the study.	645	NI	30.70
Terpenning et al. 1994	USA	1990	Cross-sectional	Hospital-based	Consecutive patients	all patients in the unit were evaluated with regard to demographics, diagnoses,clinical factors, nutritional parameters, and infection with methicillin-resistant Staphylococcus aureus, high level gentamicin-resistant enterococci, and gentamicin and/or ceftriaxone-resistant Gram-negative bacilli.	551	64.40 ± 0.50	98.37
Wu et al. 2014	China	2010	Cross-sectional	Hospital-based	Consecutive patients	A study was conducted at medical wards of National Cheng-Kung University Hospital, Douliu branch of NCKUH, and Tainan Hospital, Department of Health, Executive Yuan, at southern Taiwan between August 2009 and January 2012 Hospitalized patients were enrolled if they met the following criteria: they were older than 18 years, admitted through the emergency department and diagnosed as having Co-urinary tract infection due to one species of Enterobacteriaceae.	136	67.00	31.62
Zhang et al. 2019	China	2016	Cross-sectional	Hospital-based	Consecutive patients	Individuals in Ruijin Hospital from April 21, 2014 to December 31, 2017.	365	66.60 ± 1.40	67.67

NI: No information available.

Supplementary Table 7: Characteristics of case-control reports (expanded from Table 2 in the main paper)

Author	Country	Year of data collection	Sample based	Participant selection		Description		Sample size		Age		% men	
				Case	Control	Case	Control	Case	Control	Case	Control	Case	Control
Anesi et al. 2016	USA	2012	Hospital-based	Consecu-tive patients	Random	Case patients were defined as those with an Enterobacteriaceae urinary tract infection demonstrating resistance to an *Extended-spectrum cephalosporin resistance (ie, ceftriaxone* or cefotaxime minimum inhibitory concentration >1 µg/mL) in accordance with recent Clinical and Laboratory Standards Institute criteria.	Control patients were those who had a urinary tract infection with Extended-spectrum cephalosporin-susceptible Enterobacteriaceae during the study period (ie, ceftriaxone and cefotaxime minimum inhibitory concentrations ≤1 µg/mL). They were randomly selected and matched with case patients on study year in a 1:1 ratio.	151	151	58.67 ± 17.96	46.33 ± 28.44	28.48	12.58
Apisarnthanarak et al. 2007	Thailand	2004	Hospital-based	Random	Random	Adult patients that were infected with extended-spectrum β-lactamase producing *Escherichia coli*.	Adult patients that were infected with non extended-spectrum β-lactamase-producing *Escherichia coli*.	46	46	58.25 ± 16.51	56.5 ± 14.47	28.26	28.26
Aswani et al. 2014	India	2011	Hospital-based	NI	NI	Patients with culture positive urinary tract infection with a history of diabetes and those who were on treatment.	Patients with culture positive urinary tract infection admitted in hospital with comparable age and sex with no history of diabetes and fasting blood sugar < 110mg/dl.	181	124	60.2 ± 13.76	53.47 ± 18.56	45.86	41.94
Borer et al. 2012	Israel	2007	Hospital-based	Consecu-tive patients	Not clear	Patients colonized with carbapenem-resistant *Klebsiella pneumoniae* who developed an infection. Patients had a median age of 71 (range: 19 to 91).	Patients colonized with carbapenem-resistant *Klebsiella pneumoniae* who did not develop an infection. Patients had a median age of 72.5 (range: 21 to 95).	42	464	60.7 ± 55.30	62.8 ± 55	NI	NI
Briongos-Figuero et al. 2012	España	2010	Hospital-based	NI	Random	Hospitalised patients infected by extended-spectrum β-lactamase-producing *Escherichia coli*.	Hospitalised patients with urinary tract infection caused by non-extended-spectrum β-lactamase-producing *Escherichia coli*.	97	103	78 ± 14	77 ± 13	45.40	31.10
Chitnis et al. 2012	USA	2010	Hospital-based	Consecu-tive patients	Not clear	Patients with a Carbapenem-resistant enterobacteriaceae that were treated at a long-term	Patients without a Carbapenem-resistant enterobacteriaceae infection	34	34	68.67 ± 34.80	69.67 ± 42.56	38.00	65.00

						acute care hospital (age range: 43 - 88).	that were treated at a long-term acute care hospital (age range: 43 - 98).						
Colodner et al. 2008	Israel	2005	Hospital-based	Consecutive patients	Consecutive patients	Patients with community-acquired urinary tract infections resistance to ciprofloxacin and ofloxacin (age range: 19 - 94)	Patients with community-acquired urinary tract infections susceptible to ciprofloxacin and ofloxacin.	150	150	63.75 ± 14.20	56 ± 14	24.70	10.70
Colodner et al. 2004	Israel	NI	Hospital-based	Consecutive patients	Consecutive patients	Nonhospitalized patients with community-acquired urinary tract infection due to *Escherichia coli or Klebsiella spp.* who had extended-spectrum β-lactamase-positive strains.	Patients with urine cultures that grew extended-spectrum β-lactamase-negative *Escherichia coli* or *Klebsiella spp.* Strains.	128	183	61.5 ± 22.90	46.4 ± 24.90	35.90	13.70
Dan et al. 2016	USA	2012	Hospital-based	Consecutive patients	Consecutive patients	Patients older than 18 years with bloodstream infection due to aerobic Gram-negative bacilli (Fluoroquinolone resistant) who attended to Palmetto Health (age range: 58 to 77).	Patients older than 18 years with bloodstream infection due to aerobic Gram-negative bacilli (Fluoroquinolone sensible) who attended to Palmetto Health (age range: 53 to 78).	143	681	67.7 ± 14.23	65.3 ± 18.60	59.00	44.00
Dodds Ashley et al. 2004	USA	1997	Hospital-based	Consecutive patients	Consecutive patients	Patients who developed Postoperative Mediastinitis due to methicillin-resistant *Staphylococcus aureus*.	Patients who developed Postoperative Mediastinitis due to methicillin-sensitive Staphylococcus aureus.	64	79	67.30	61.80	45.30	69.60
García-Tello et al. 2017	Spain	2012	Hospital-based	Consecutive patients	Consecutive patients	Symptomatic patients with isolates of extended spectrum beta-lactamase-producing Enterobacteriaceae who had been hospitalized or admitted to the emergency room.	Patients with isolates of non-extended spectrum beta-lactamase-producing Enterobacteriaceae who had been hospitalized or admitted to the emergency room.	416	1108	72.60 ± 20.50	54.70 ± 29.80	36.10	21.50
Hayakawa et al. 2013	USA	2009	Hospital-based	NI	NI	Patients who had clinical isolates of vancomycin resistant *Enterococcus faecalis*.	Patients with isolates of vancomycin-susceptible E. faecalis. Matching parameters for cases included (i) anatomic site of vancomycin resistant Enterococcus isolation, (ii) hospital or outpatient facility where the patient was cared for, (iii) unit or clinic from which vancomycin resistant Enterococcus was recovered,	532	532	66.00 ± 16.50	62.40 ± 18.10	45.30	48.70

							(iv) calendar year, and (v) time at risk (i.e., time from admission to culture for patients with enterococci). Time at risk for the vancomicyn sensible *E. faecalis* case had to be at least as long as the time at risk for the matched vancomycin resistant Enterococcus.						
Hershow et al. 1998	USA	1990	Hospital-based	Ni	NI	Inpatients with nosocomially acquired ciprofloxacin resistant methicillin-resistant *Staphylococus aureus* infection.	Inpatients with nosocomially acquired ciprofloxacin susceptible methicillin-resistant *Staphylococus aureus* infection	15	10	54.00	44.00	53.00	30.00
Hershow et al. 1998	USA	1990	Hospital-based	Ni	NI	Inpatients with nosocomially acquired ciprofloxacin resistant methicillin-resistant *Staphylococus aureus* infection	Inpatients with nosocomially acquired ciprofloxacin susceptible methicillin-resistant *Staphylococus aureus* infection	56	14	50.00	57.00	48.21	35.71
Hsu et al. 2005	USA	2001	Hospital-based	NI	NI	Fluoroquinolone-resistant Pseudomonas aeruginosa hospitalized adult patients	Fluoroquinolone-susceptible *Pseudomonas aeruginosa* hospitalized adult patients	91	86	73.8 ± 15.20	68 ± 17.40	43.00	49.00
Isendhal et al. 2019	Sweden	2009	Hospital-based	Consecu-tive patients	Random	Patients with bloodstream infection with extended-spectrum b-lactamase-producing Enterobacteriaceae [resistant to cefotaxime and/or ceftazidime but inhibited by clavulanic acid or cloxacillin (in the latter case mostly followed by PCR to confirm the presence of plasmid-mediated AmpC)]	Controls randomly selected from the Swedish population using the Total Population Register. Up to 10 controls per case were included matched on age in years, sex and residence county.	945	9390	65.62 ± 15.7		58.90	
Jinnah et al. 1996	Bangladesh	NI	Hospital-based	Random selection	Random selection	Diabetic out-patients with urinary tract infection of either sex	Non diabetic out-patients with urinary tract infection of either sex	150	150	NI	NI	NI	NI
Khurram et al. 2007	Pakistan	1998	Hospital-based	Consecu-tive patients	Consecu-tive patients	Patients hospitalized at intense care unit in the Aga Khan University Hospital Cases who had a clinically significant infection with methicillin-	Patients hospitalized at intense care unit in the Aga Khan University Hospital Cases who remained colonized with methicillin-	37	20	52.8 ± 15.10	34.9 ± 21.20	54.10	65.00

Carrillo-Larco RM, *et al. J Epidemiol Community Health* 2021; 76:75–84. doi: 10.1136/jech-2020-216029

						resistant *Staphylococcus aureus* at any time during their intense care unit stay.	resistant Staphylococcus aureus but did not develop infection at any time during their intense care unit stay. Colonization was defined as the isolation of methicillin-resistant *Staphylococcus aureus,* documented by its growth in culture (culture positivity), from non-sterile sites in the absence of signs and symptoms of the associated disease.						
Kim et al. 2014	Korea	2008	Hospital-based	Consecu-tive patients	Consecu-tive patients	Patients admitted to intense care unit at tertiary teaching centres in Korea with hospital acquired pneumonia (HAP) caused by carbapenem-resistant Gram-negative bacteria.	Patients admitted to intense care unit at tertiary teaching centres in Korea with hospital acquired pneumonia caused only by carbapenem-sensible Gram-negative bacteria.	82	122	66.3 ± 10.50	67.3 ± 15.70	59.80	68.90
Manzur et al. 2007	Spain	1997	Hospital-based	Consecu-tive patients	Consecu-tive patients	Patients who attended to a tertiary-care teaching centre in Spain from January 1991 to December 2003, with clinically significant methicillin-resistant Staphylococcus aureus-bloodstream infection diagnosed within 48 h of admission. Around 80% of the cases had at least 60 years, 46% were males.	Patients who attended to a tertiary-care teaching centre in Spain from January 1991 to December 2003, with methicillin-sensible Staphylococcus aureus-bloodstream infection. Around 57% of the cases had at least 60 years, 43% were males.	50	98	69.06 ± 13.90	58.33 ± 18.24	45.00	65.30
Park et al. 2015	Korea	2007-2013	Hospital-based	Consecu-tive patients	Random	Patients >15 year of age diagnosed with community acquired acute pyelonephritis caused by extended-spectrum β-lactamase-Escherichia coli. For patients with multiple episodes, only the first episode was included in the analysis	Patients >15 year of age diagnosed with community acquired acute pyelonephritis caused by non-extended-spectrum β-lactamase-Escherichia coli	75	225	NI	NI	12.00	6.20
Ren et al. 2019	China	2015	Hospital-based	Consecu-tive patients	Not clear	Patients at hospital intense care unit ward with diagnosis of pan-resistant Acinetobacter baumannii pneumonia between	Patients with Acinetobacter baumannii pneumonia were selected in our hospital intense care unit ward, these	98	49	NI	NI	NI	NI

						January 2013 to February 2016.	were non-highly resistant patients with hospital-acquired pneumonia diagnostic criteria.						
Saade et al. 2016	USA	2000-2013	Hospital-based	NI	NI	Patients undergoing TBP with positive urine or blood cultures for Escherichia coli within 30 days after TBP, and determined if the Escherichia coli isolates were resistant to fluoroquinolones.	Patients undergoing TBP with positive urine or blood cultures for Escherichia coli within 30 days after TBP, and determined if the Escherichia coli isolates were sensitive to fluoroquinolones.	428	59041	62.95 ± 12.10	66.75 ± 5.76	100	100
Saibal et al. 2012	Bangladesh	2009	Hospital-based	Convenient and purposive	Convenient and purposive	Diabetic hospitalized adults (18-80 years old) patients with community acquired pneumonia	Non diabetic hospitalized adults (18-80 years old) patients with community acquired pneumonia	47	43	56.3 ± 12.20	35.7 ± 10.50	68.10	72.10
Silva et al. 2006	Brazil	2002	Hospital-based	Consecutive patients	Consecutive patients	Patients with blood or urinary tract cultures hospitalized at a private tertiary hospital in Brazil who had infections with extended-spectrum β-lactamase producer bacteria.	Patients with blood or urinary tract cultures hospitalized at a private tertiary hospital in Brazil who had infections with non-extended-spectrum β-lactamase producer bacteria.	56	51	65.80	61.50		
Soraas et al. 2013	Norway	2010	Hospital-based	Consecutive patients	Random	Patietns that lived in the South-Eastern part of Norway who were willing to participate and had a community acquired extended-spectrum β-lactamase-positive urinary tract infection.	Patietns that lived in the South-Eastern part of Norway who were willing to participate and had a community acquired extended-spectrum β-lactamase-negative urinary tract infection.	100	190	55.00 ± 19.00	64.00 ± 17.00	12.00	12.00
Vinken et al. 2018	Netherlands	2014	Hospital-based	Consecutive patients	NI	Female outpatients ≥30 years old with T2DM and a positive urine culture	Non-diabetes female outpatient with a positive urine culture matched by sex, age category and general practitioner with cases	283	283	73.30 ± 12.10	72.20 ± 13.10	0.00	0.00
Wright et al. 2000	USA	1996	Hospital-based	Consecutive patients	Consecutive patients	Patients at least 16 years of age who were treated in the Vanderbilt University Hospital emergency department between January 1995 and March 1997 who had positive cultures for multi-drug resistance bacteria.	Patients at least 16 years of age who were treated in the Vanderbilt University Hospital emergency department between January 1995 and March 1997 who had positive cultures for non multi-drug resistance bacteria.	118	317	NI	NI	30.50	15.10

Wright et al. 1999	USA	1996	Hospital-based	Consecutive patients	Consecutive patients	Patients who attended the emergency department with urinary tract infections caused by coliforms that were resistant to trimethoprim-sulfamethoxazole.	Patients who attended the emergency department with urinary tract infections caused by coliforms that were sensible to trimethoprim-sulfamethoxazole.	67	381	NI	NI	17.90	16.00

Supplementary Table 8: Adjusted association estimates of antibiotic-resistant from cross-sectional and cohort reports (expanded from Table 3 in the main paper)

Infection site	Author	Antibiotic	Bacteria	Measure of association	Adjustement
Urinary tract infection	Chen et al. 2013	Cefazolin	*Escherichia coli* (69%), *K. pneumoniae* (7%), *Pseudomonas aeruginosa (5%), Proteus mirabilis (4%), Enterobacter* (3%) *and Citrobacter species* (2%).	OR: 2.32 [1.32 ; 4.07]	Sex
	Chiu et al. 2017	Cefazolin	*Escherichia coli* (54.5%) *was most commonly found. Other common uropathogens were Klebsiella pneumoniae* (13.1%)*; Enterococcus spp.* (7.1%), *Pseudomonas aeruginosa (4.6%), and Proteus mirabilis* (3.5%).	OR: 4.17 [2.0 ; 9.09]	Age 65 y; Male gender; Residents of health care facility; benign prostate hypertrophy; urinary tract infection within 1 y; NG tube; Dysuria; frequency/urgency; Temperature ≥ 38.3 °C.
	Ho et al. 2019	Amoxicilin-clavunate	*Escherichia coli, Klebsiella sp., Proteus mirabilis, Enterobacteriaceae,* or *Staphylococcus saprophyticus* only.	OR: 2.54 [1.09 ; 5.88]	Gender; Genitourinary abnormalities; Antibiotic given and susceptibility (vs no antibiotic): Given amoxicillin-clavulanate; susceptible; Given other antibiotic; susceptible.
	Wu et al. 2014	Levofloxacin	*Enterobacteriaceae pathogens (Escherichia coli ; Klebsiella pneumoniae ; Proteus spp. and Klebsiella oxytoca ; E. cloecae; K. oxytoca; M. morganil.; S. marcescens.; P. vulgaris.; Citrobacter).*	OR: 3.80 [1.50 ; 9.90]	Age; gender; recurrent urinary tract infection; prior hospitalization in the past 6 mo; prior antibiotic in the past 60d; urinary function abnormality; indwelling urinary catheter; old stroke; altered consciousness; urinary symptoms; chills; fever; haematuria.
Respiratory tract infection	Madaras et al. 2012	Non-pseudomonal third generation cephalosporins (ceftriaxone or cefotaxime) or non-pseudomonal 8-methoxy fluoroquinolones (moxifloxacin; gatifloxacin); the VA preferred agents for	Methicillin-resistant *Staphylococcus aureus*	OR: 2.20 [1.20 ; 4.30]	Nursing home residence or discharge ≤180 days prior to admission; positive methicillin-resistant *Staphylococcus aureus* status prior to admission; Anti-pseudomonal fluoroquinolone exposure ≤365

		treatment of community-acquired pneumonia			days prior to admission; 3rd generation cephalosporin exposure ≤365 days prior to admission; Chronic inhaled corticosteroids.
	Madaras et al. 2012	Non-pseudomonal third generation cephalosporins (ceftriaxone or cefotaxime) or non-pseudomonal 8-methoxy fluoroquinolones (moxifloxacin; gatifloxacin); the VA preferred agents for treatment of CAP	*Methicillin-resistant Staphylococcus aureus* and *Pseudomonas aeruginosa*	OR: 1.70 [1.00 ; 2.80]	Nursing home residence or discharge ≤180 days prior to admission; positive methicillin-resistant Staphylococcus aureus status prior to admission; cephalosporin exposure ≤365 days prior to admission; infusion therapy ≤30 days prior to admission; direct intense care unit admission upon hospitalization.
	Micek et al. 2015	Aminoglycosides; antipseudomonal carbapenems; antipseudomonal cephalosporins; antipseudomonal fluoroquinolones; antipseudomonal penicillins plus β-lactamase inhibitors; monobactams; phosphonic acids; and polymixins	*Pseudomonas aeruginosa*	OR: 1.90 [1.21 ; 3.00]	Age; sex; residence in a community settings prior admission; residence in an inpatient rehabilitation facility prior to admission; antibiotics in the previous 30 days; COPD; solid tumour; dementia; intense care unit admission.
Complicated Skin and Skin Structure Infections	Jaaskelainen et al. 2017	Carbapenem; piperacillin-tazobactam	*Methicillin-sensitive Staphylococcus aureus; Streptococcus pyogenes; Streptococcus agalactiae; β-hemolytic streptococci; Streptococcus pneumoniae; α-hemolytic streptococci; Enterobacteriacae; Pseudomonas;* Anaerobic bacteria*; Enterococci;* others	OR: 1.67 [0.96 ; 2.91]	Age; chronic renal failure; respiratory disease; Injection drug abuse; Abscess; cellulitis/fasciitis; Number of days between symptoms Start and diagnosis.
	Jaaskelainen et al. 2017	Cefadroxil; cefotaxim; ceftriaxone; cefuroxime; cephalexin.	*Methicillin-sensitive Staphylococcus aureus; Streptococcus pyogenes; Streptococcus agalactiae; β-hemolytic streptococci; Streptococcus pneumoniae; α-*	OR: 1.07 [0.69 ; 1.64]	Age; chronic renal failure; respiratory disease; injection drug abuse; abscess; cellulitis/fasciitis; number of days between symptoms start and diagnosis.

			hemolytic streptococci; Enterobacteriaceae; Pseudomonas; Anaerobic bacteria*; Enterococi;* others		
	Jaaskelainen et al. 2017	Amoxicillin; benzylpenicillin; phenoxymethylpenicillin.	*Methicillin-sensitive Staphylococcus aureus; Streptococcus pyogenes; Streptococcus agalactiae; β-hemolytic streptococci; Streptococcus pneumoniae; α-hemolytic streptococci; Enterobacteriaceae; Pseudomonas;* Anaerobic bacteria*; Enterococi;* others	OR: 0.94 [0.46 ; 1.91]	Age; chronic renal failure; respiratory disease; injection drug abuse; abscess; cellulitis/fasciitis; number of days between symptoms start and diagnosis.
	Jaaskelainen et al. 2017	Clindamycin; doxycyclin; fluoroquinolone; fusidic acid; linezolid; metronidazole; cotrimoxazole; tobramycin; vancomycin.	*Methicillin-sensitive Staphylococcus aureus; Streptococcus pyogenes; Streptococcus agalactiae; β-hemolytic streptococci; Streptococcus pneumoniae; α-hemolytic streptococci; Enterobacteriaceae; Pseudomonas;* Anaerobic bacteria*; Enterococi;* others	OR: 0.79 [0.38 ; 1.64]	Age; chronic renal failure; respiratory disease; injection drug abuse; abscess; cellulitis/fasciitis; number of days between symptoms start and diagnosis.
	Jaaskelainen et al. 2017	Cloxacillin; flucloxacillin; other β-lactamase-stable penicillins	*Methicillin-sensitive Staphylococcus aureus; Streptococcus pyogenes; Streptococcus agalactiae; β-hemolytic streptococci; Streptococcus pneumoniae; α-hemolytic streptococci; Enterobacteriaceae; Pseudomonas;* Anaerobic bacteria*; Enterococi;* others	OR: 0.50 [0.24 ; 1.08]	Age; chronic renal failure; respiratory disease; injection drug abuse; abscess; cellulitis/fasciitis; number of days between symptoms start and diagnosis.
